# Supplementary material for: Exosomal miRNA-155-5p from M1-polarized macrophages suppresses angiogenesis by targeting GDF6 to interrupt diabetic wound healing
Source: Mol Ther Nucleic Acids. 2023 Nov 10;34:102074. doi: 10.1016/j.omtn.2023.102074 (PMC10701080; doi:10.1016/j.omtn.2023.102074)
Supplement: Document S1. Figures S1–S4 and Table S2 [file mmc1.pdf]

OMTN, Volume *34*

## Supplemental information

# Exosomal miRNA-155-5p from M1-polarized macrophages suppresses angiogenesis by targeting GDF6 to interrupt diabetic wound healing

**Ruohan Lou, Jiali Chen, Fei Zhou, Tian Zhang, Xiuping Chen, Chunming Wang, Bing Guo, and Ligen Lin**

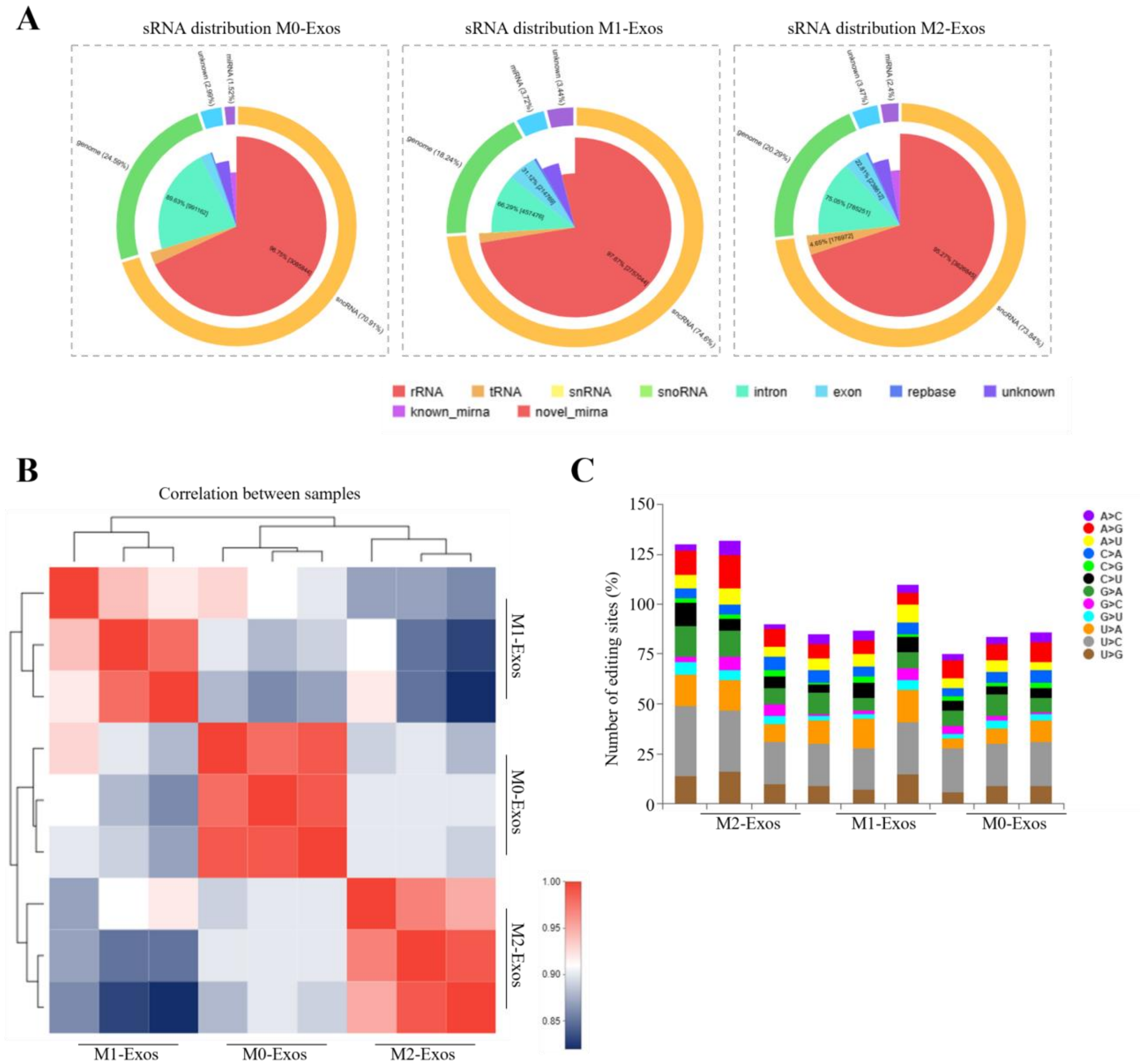


**Figure S1.** Bioinformatics analysis of exosomal miRNAs. (A) sRNA distribution in exosomes from different groups of mice. (B) Heat map of correlation between samples. (C) Distribution map of miRNA base editing types.

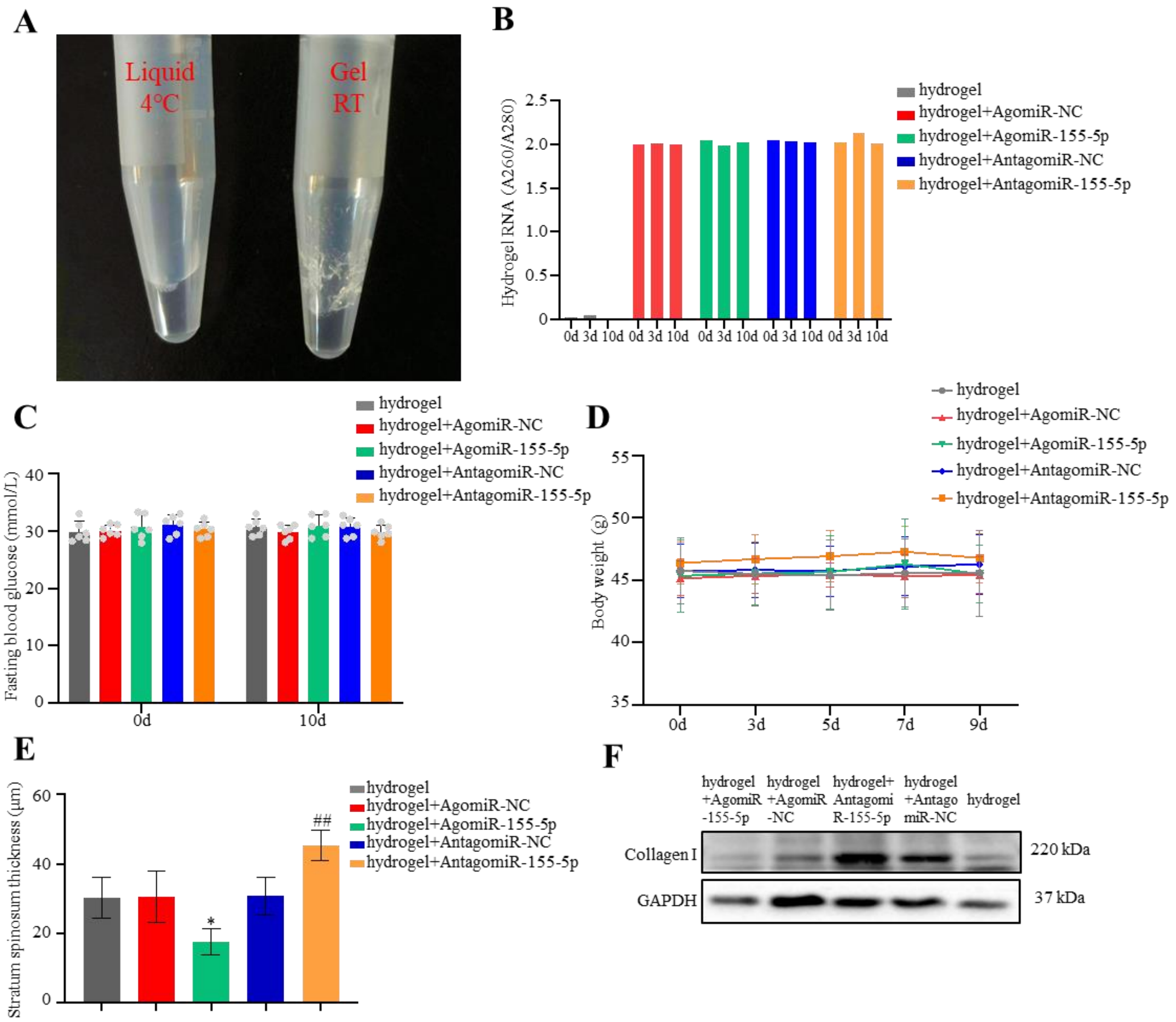


**Figure S2.** Hydrogel preparation and animal experiments. (A) 25% Pluronic F-127 solution is a liquid at 4 ℃ and a gel at room temperature. (B) RNA purity in hydrogel. (C) Blood glucose level of *db/db* mice on day 0 and day 10. (D) Body weight of *db/db* mice. (E) Stratum spinosum thickness in wound tissue. (F) The protein expression of collagen I in wound tissue. GAPDH was used as a loading control. $^{*}p < 0.05$, AgomiR-155-5p *vs.* AgomiR-NC; $^{\#\#}p < 0.01$, AntagomiR-155-5p *vs.* AntagomiR-NC.

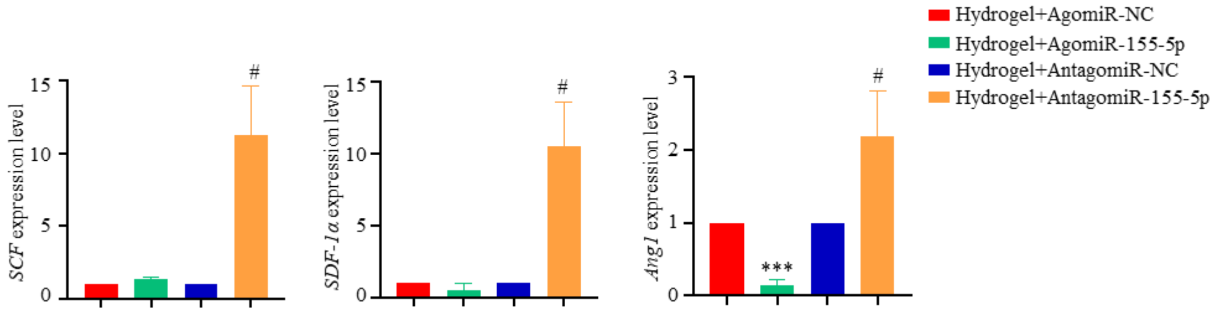


**Figure S3.** The mRNA expression levels of angiogenic genes in wound tissues were analyzed by q-RT-PCR. ***$p < 0.001$, AgomiR-155-5p *vs.* AgomiR-NC; # $p < 0.05$, AntagomiR-155-5p *vs.* AntagomiR-NC.

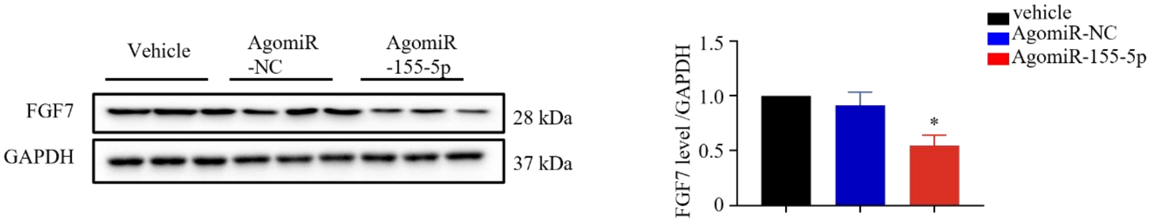


**Figure S4.** The protein expression of FGF7 in wound tissues. GAPDH was used as a loading control. Data are expressed as means ± SD. $^{*}p < 0.05$, AgomiR-155-5p *vs.* AgomiR-NC.

**Table S1**

Levels of BMDM-Exos miRNAs and top 10 differentially expressed miRNAs in M1-Exos and M2-Exos.

**Table S2**

Primers used for RT-PCR.

| Names of microRNAs and genes | Primer sequence (5′ to 3′) |
|---|---|
| has-β-actin-forward | AGAGCTACGAGCTGCCTGAC |
| has-β-actin-reverse | AGCACTGTGTTGGCGTACAG |
| has-Bcl-2-forward | GATAACGGAGGCTGGGATGC |
| has-Bcl-2-reverse | TCACTTGTGGCCCAGATAGG |
| has-Bax-forward | CCCTTTTGCTTCAGGGTTTC |
| has-Bax-reverse | GAGACACTCGCTCAGCTTCTTG |
| hsa-CyclinD1-forward | TTGCCCTCTGTGCCACAGAT |
| hsa-CyclinD1-reverse | TCAGGTTCAGGCCTTGCACT |
| hsa-CyclinD3-forward | TACCCGCCATCCATGATCG |
| hsa-CyclinD3-reverse | AGGCAGTCCACTTCAGTGC |
| hsa-miRNA-155-5p-RT | CTCAACTGGTGTCGTGGAGTCGGCAATTCAGTTGAGAACCCCTA |
| has-miRNA-155-5p-forward | GCCGAGTTAATGCTAATCGTG |
| has-miRNA-155-5p-reverse | CTCAACTGGTGTCGTGGA |
