# Supplementary material for: Exosomal miRNA-155-5p from M1-polarized macrophages suppresses angiogenesis by targeting GDF6 to interrupt diabetic wound healing
Source: Mol Ther Nucleic Acids. 2023 Nov 10;34:102074. doi: 10.1016/j.omtn.2023.102074 (PMC10701080; doi:10.1016/j.omtn.2023.102074)
Supplement: Document S2. Article plus supplemental information [file mmc3.pdf]

Molecular Therapy
Nucleic Acids
Original Article

Exosomal miRNA-155-5p from M1-polarized macrophages suppresses angiogenesis by targeting GDF6 to interrupt diabetic wound healing

Ruohan Lou,[1] Jiali Chen,[1] Fei Zhou,[1] Tian Zhang,[2] Xiuping Chen,[1,3] Chunming Wang,[1,3] Bing Guo,[2] and Ligen Lin[1,3]

[1]State Key Laboratory of Quality Research in Chinese Medicine, Institute of Chinese Medical Sciences, University of Macau, Taipa, Macau 999078, China; [2]Guizhou Provincial Key Laboratory of Pathogenesis and Drug Research on Common Chronic Diseases, Guizhou Medical University, Guiyang 550025, China; [3]Department of Pharmaceutical Sciences and Technology, Faculty of Health Sciences, University of Macau, Taipa, Macau 999078, China

Unprogrammed macrophage polarization, especially prolonged activation of proinflammatory macrophages, is associated with delayed wound healing in diabetic objectives. Macrophage-derived exosomes cargo a variety of microRNAs (miRNAs), participating in different stages in wound healing. Here, exosomes were isolated from naive bone marrow–derived macrophages (BMDMs) (M0-Exos), interferon-γ plus lipopolysaccharide-polarized BMDMs (M1-Exos), and interleukin-4-polarized BMDMs (M2-Exos). M1-Exos impaired migration and tube formation in human umbilical vein endothelial cells (HUVECs) compared to M0-Exos, whereas M2-Exos exhibited the opposite effects. High-throughput sequencing was performed to decipher the miRNA expression profiles in M0-Exos, M1-Exos, and M2-Exos. A total of 63 miRNAs were identified to be differentially expressed in exosomes derived from polarized BMDMs. Among them, miRNA-155-5p is highly expressed in M1-Exos, which interrupted angiogenesis in HUVECs. Furthermore, miRNA-155-5p directly binds to the 3′ UTR of growth differentiation factor 6 (*GDF6*) mRNA to suppress its protein expression. Lastly, local administration of a temperature-sensitive hydrogel Pluronic F-127 loading miRNA-155-5p antagomiR promoted angiogenesis and accelerated wound healing in diabetic *db/db* mice via enhancing GDF6. In summary, this study deciphered the miRNA expression profiles in exosomes from polarized macrophages. M2-like macrophage-derived exosomes and miRNA-155-5p inhibitors could be promising therapeutics against diabetic foot ulcers.

INTRODUCTION

Diabetes is a chronic metabolic disorder with a series of severe complications. Among them, impaired wound healing, particularly diabetic foot ulcers (DFUs), leads to significant morbidity, which ultimately causes nontraumatic limb amputation.[1] The risk of developing DFUs for diabetic patients across their lifetime has been estimated to be 30%.[2] Unfortunately, no effective medical tools for DFUs are available in clinic. Wound healing is a complex process involving a series of coordinated and overlapping stages, including coagulation, inflammation, angiogenesis, and remodeling of the cell matrix. Delay or stagnation in any of these steps causes impaired wound healing.[3] During wound healing, macrophages clear pathogens and regulate the proliferative phase of the repair process directly or indirectly through paracrine modes.[4] In diabetic patients and mice, about 80% cells at the edge of chronic wounds are proinflammatory M1 macrophages, and the transition to an anti-inflammatory M2-like phenotype does not occur in time.[5] The M2-like macrophages regulate tissue remodeling and inflammation resolution, which underpin their critical role in maintaining microenvironmental homeostasis in diabetic wounds. Therefore, the impaired macrophage polarization is one of the main causes of healing stagnation in the inflammatory phase and poor wound healing in diabetic patients.

Exosomes are extracellular nanovesicles with a diameter of 30 to 100 nm, which mediate universal cell-to-cell communication by transferring specific components to recipient cells, including proteins, lipids, and nucleic acids.[6] Emerging evidence suggests that exosomes play a key role in regulating matrix microenvironment homeostasis.[7] In diabetic mice, M1 macrophages at the wound site directly transform into M2 macrophages after subcutaneous administration of M2 macrophage-derived exosomes, which accelerates wound healing by enhancing angiogenesis and epithelial regeneration.[8]

MicroRNAs (miRNAs) are a class of 17- to 25-nt small noncoding RNAs (ncRNAs) that regulate posttranscriptional gene silencing by binding to the 3′ UTR or open reading frame region of target mRNAs.[9] miRNAs stably exist in body fluids and can be packaged into exosomes or microvesicles to protect them from degradation. Given the transportability of vesicles, the information transmission through vesicles is a promising way of cell-to-cell communication.[10] Therefore, exosomal miRNAs from polarized macrophages may participate in the regulation of diabetic wound healing.

Received 30 August 2023; accepted 8 November 2023;
https://doi.org/10.1016/j.omtn.2023.102074.

Correspondence: Ligen Lin, State Key Laboratory of Quality Research in Chinese Medicine, Institute of Chinese Medical Sciences, University of Macau, Taipa, Macau 999078, China.
E-mail: ligenl@um.edu.mo

This is an open access article under the CC BY license (http://creativecommons.org/licenses/by/4.0/).

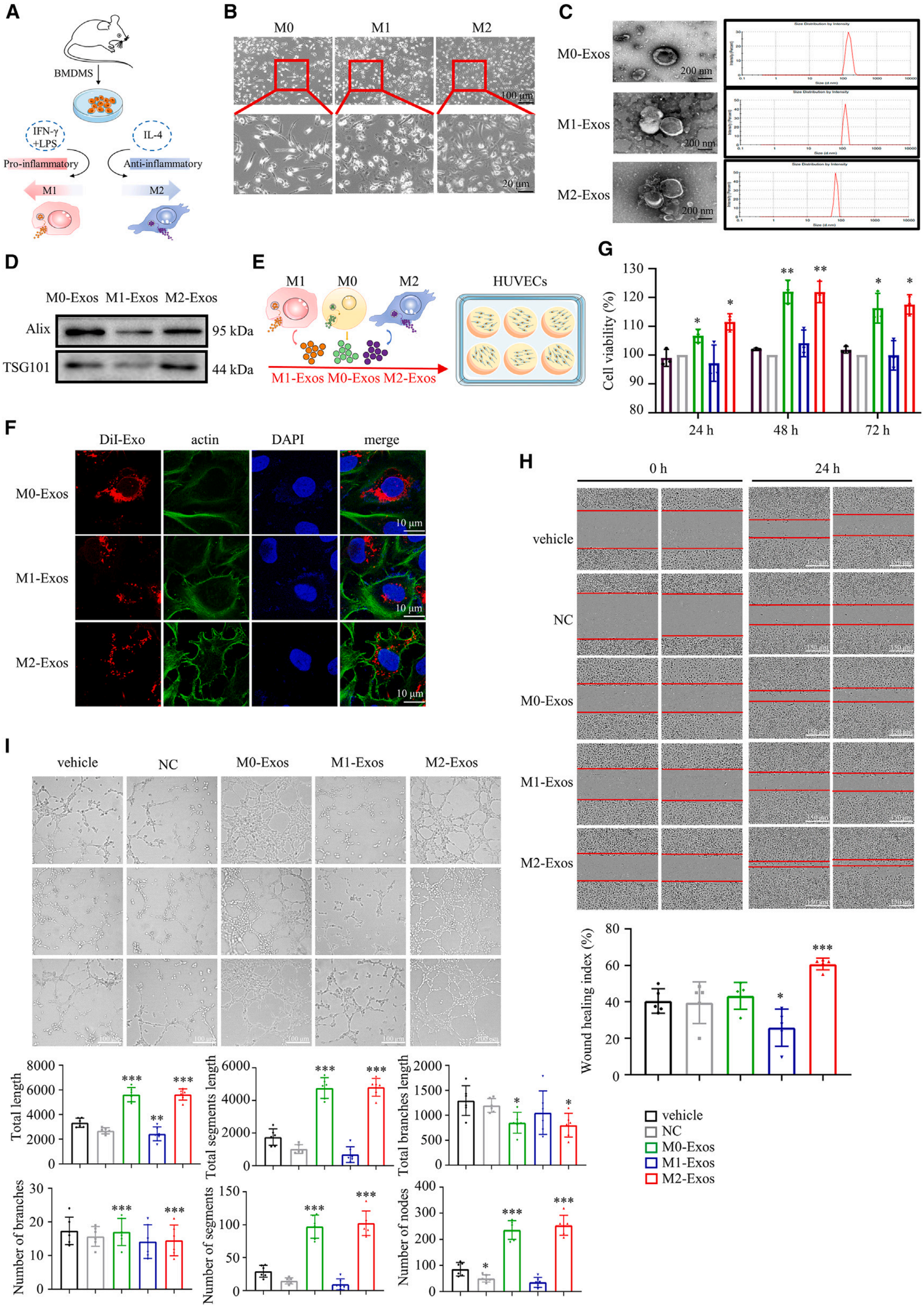

(legend on next page)

Here, we deciphered the miRNA expression profiles in exosomes derived from polarized bone marrow–derived macrophages (BMDMs), either proinflammatory M1-like or anti-inflammatory M2-like. Among the differentially expressed miRNAs, miRNA-155-5p, highly enriched in exosomes from M1-like macrophages, was identified as interrupting endothelial cell function and angiogenesis, and the miRNA-155-5p antagomiR was found to accelerate wound healing in diabetic *db/db* mice.

RESULTS

BMDMs-derived exosomes modulate angiogenesis in HUVECs

Macrophages orchestrated vessel sprouting and regeneration in wound angiogenesis. BMDMs can differentiate into mature macrophages (M0) in the presence of growth factors and other signaling molecules, which can be further induced to proinflammatory M1 macrophages by a variety of stimuli such as lipopolysaccharides (LPS), interferon-γ (IFN-γ), and pathogen-associated molecular patterns, or M2 anti-inflammatory macrophages under stimuli such as interleukin-4 (IL-4).[11] To disclose their roles in mediating cellular interaction and manipulating angiogenesis, BMDM-derived exosomes were first isolated. To obtain BMDM-derived exosomes, bone marrow–derived stem cells (BMSCs) were isolated from the femurs and tibias of C57BL/6J mice, differentiated into naive BMDMs (M0-BMDMs), and then induced to M1-BMDMs and M2-BMDMs by LPS plus IFN-γ and IL-4, respectively (Figure 1A). Under the microscope, the M1-BMDMs showed characteristic fried egg shapes, whereas the M2-BMDMs showed mixed populations of fried eggs and spindles (Figure 1B), which were consistent with a previous report.[12] Next, exosomes were isolated from M0-BMDMs (M0-Exos), M1-BMDMs (M1-Exos) and M2-BMDMs (M2-Exos). Transmission electron microscopy (TEM) images showed that the exosomes derived from three types of BMDMs showed a standard bowl-shaped vesicle morphology, and particle size distribution results suggested these exosomes as having a diameter ranging from 80 to 150 nm (Figure 1C). The protein expression of the exosomal markers Alix and TSG101 further confirmed the successful isolation of exosomes (Figure 1D). Collectively, the above evidence indicated that exosomes were successfully isolated from BMDMs with characteristic morphology and similar size.

Next, human umbilical vein endothelial cells (HUVECs) were incubated in serum-free medium supplied with 50 μg/mL exosomes from different types of BMDMs (Figure 1E). The confocal images showed that BMDM-derived exosomes could be indiscriminately ingested by HUVECs within 7 h (Figure 1F). Interestingly, M0-Exos and M2-Exos significantly increased the proliferation of HUVECs, but not M1-Exos, assessed by Cell Counting Kit 8 (CCK-8) assay (Figure 1G). As anticipated, the migration potentials were significantly enhanced in HUVECs treated with M0-Exos or M2-Exos, but not those treated with M1-Exos, assessed by cell scratch assay (Figure 1H). Next, the tube formation assay was conducted. Quantitative analysis of key parameters in tube formation including the total length, total segments length, total branches length, number of branches, number of segments, and number of nodes showed that the treatment of M0-Exos or M2-Exos promoted angiogenesis, but not M1-Exos (Figure 1I). Taken together, BMDMs-derived exosomes can be taken up by HUVECs, and M2-BMDM- or M0-BMDM-derived exosomes promote the proliferation, migration, and tube formation of HUVECs.

miRNA expression profiles in BMDM-derived exosomes

Exosomes cargo a variety of components, including miRNAs, DNAs, proteins, and lipids. Among them, miRNAs play important roles in cellular communication through posttranscriptional patterns. To decipher the miRNA expression profiles in exosomes derived from polarized BMDMs, miRNA sequencing was performed. Small RNA (sRNA) distribution results showed that the known miRNAs in M0-Exos, M1-Exos, and M2-Exos accounted for 1.52%, 3.72%, and 3.47 of their total sRNAs, respectively (Figure S1A). The correlation difference between samples in the same group was negligible (Figure S1B). To identify the conserved miRNAs, all of the ncRNA reads from different types of BMDM-derived exosomes libraries were compared with the known mouse miRNAs in miRBase version 20. To focus on the highly represented miRNAs, sequences less than 10 reads were removed. Finally, 385, 338, and 384 miRNAs were identified in M0-Exos, M1-Exos, and M2-Exos, respectively. Venn analysis indicated that, among them, 330 miRNAs commonly exist in M0-Exos, M1-Exos, and M2-Exos; the numbers of miRNAs specifically existing in M0-Exos, M1-Exos, and M2-Exos were 81, 60, and 92, respectively (Figure 2A). The structural analysis of known miRNAs revealed that there are base changes in some sites of partial sequences, which may make the target genes diverse (Figure S1C). Principal-component analysis (PCA) showed that miRNAs were unambiguously segregated into three tight clusters (Figure 2B).

Next, we compared the miRNA expression levels in different types of BMDM-derived exosomes. Using a 2-fold change and $p < 0.05$ as the threshold cutoff, 63 miRNAs were identified as being differentially expressed in different types of BMDM-derived exosomes (Figure 2C). Among them, 18 miRNAs were upregulated and 20 miRNAs were

Figure 1. BMDMs-derived exosomes manipulated angiogenesis in HUVECs

(A) The experimental procedure for polarized BMDMs. (B) Morphology of polarized macrophages. Scale bar, 100 μm (top) or 20 μm (bottom). (C) The ultrastructure of exosomes observed by TEM and the average particle size distribution of exosomes. Scale bar, 200 nm. (D) The protein expression of exosome markers, including Alix and TSG101. (E) Treatment of exosomes derived from polarized macrophages on HUVECs. (F) The ingestion of exosomes into HUVECs. Exosomes were stained with red fluorescent dye DiI, and actin in HUVECs was stained with actin-Tracker Green-488. Scale bar, 10 μm. (G) The cell viability of HUVECs treated with exosomes from polarized BMDMs, assessed by CCK-8 assay. (H) The migration capacity of HUVECs treated with exosomes from polarized BMDMs, assessed by cell scratch assay. Scale bar, 100 μm. (I) The tube-formation capacity of HUVECs treated with exosomes from polarized BMDMs. Scale bar, 150 μm. Data are means ± SDs. n = 6. *p < 0.05;**p < 0.01; ***p < 0.001; Exos versus NC.

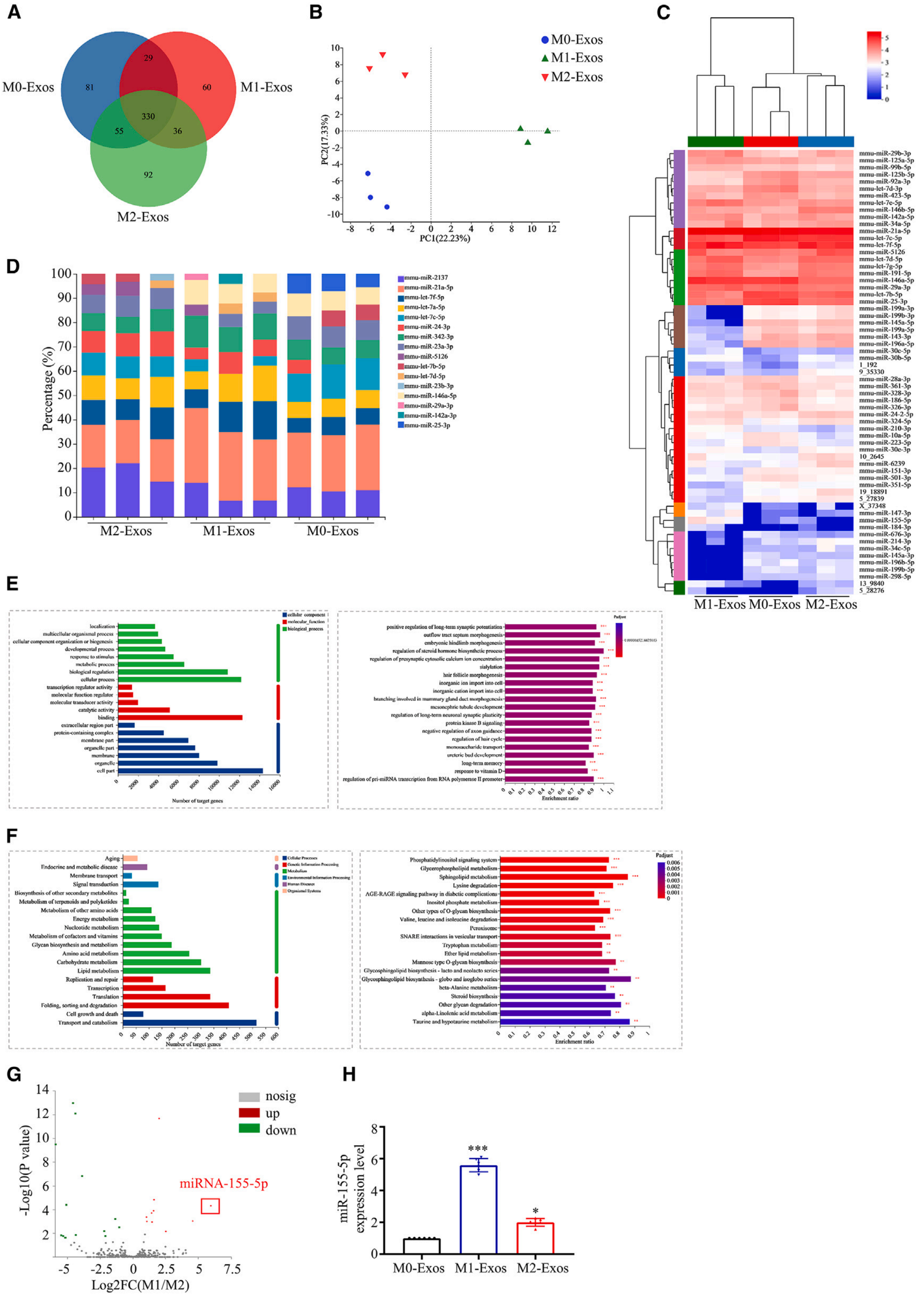

(legend on next page)

downregulated in M1-Exos, compared with M0-Exos; 16 miRNAs were upregulated and 12 miRNAs were downregulated in M2-Exos, compared with M0-Exos. The stacking diagram of expression levels showed the distribution of the top 10 miRNAs in each group (Figure 2D). To better understand the potential biological processes associated with the differences in miRNA profiles in different types of BMDM-derived exosomes, Gene Ontology (GO) and Kyoto Encyclopedia of Genes and Genomes (KEGG) analyses were performed using the online DAVID 6.8 bioinformatics resource. The GO database classifies and annotates genes and gene products in terms of their biological process, molecular function, and cellular component.[13] Among biological process terms, the differently expressed genes were mainly enriched in the regulation of cellular component biogenesis, biological regulation, cell aggregation, and extracellular part (Figure 2E). KEGG contains seven major pathways: metabolism, genetic information processing, environmental information processing, cellular processes, organismal systems, human diseases, and drug development. KEGG annotation and enrichment analysis showed that the main functions of miRNA target genes were related to cell cycle, cell growth, and cell death (Figure 2F). The analysis of miRNA expression between M1-Exos and M2-Exos showed many differently expressed miRNAs (Figure 2G; Table S1). Among them, miRNA-155-5p showed the most significant difference between M1-Exos and M2-Exos (Figure 2G; Table S1). The qRT-PCR results further supported that miRNA-155-5p is highly expressed in M1-Exos compared to M0-Exos and M2-Exos (Figure 2H). Taken together, the present study deciphered the miRNA expression profiles in different types of BMDM-derived exosomes and identified that miRNA-155-5p is highly expressed in M1-Exos.

miRNA-155-5p suppresses angiogenesis in HUVECs

To verify the role of miRNAs from polarized macrophage-derived exosomes, we next explored the effects of miRNA-155-5p on angiogenesis in HUVECs. HUVECs were transfected with negative control agomiR (NC agomiR) or miRNA-155-5p agomiR; the latter increased the miRNA-155-5p expression around 7-fold (Figure 3A). The 5-ethynyl-2′-deoxyuridine (EdU) results suggested that miRNA-155-5p agomiR suppressed the proliferation of HUVECs (Figure 3B). miRNA-155-5p agomiR decreased the expression of *Cyclin D1*, *Cyclin D3*, and *Bcl2*, and increased the expression of *Bax*, indicating that miRNA-155-5p suppressed cell proliferation and promoted apoptosis (Figure 3C). Both the transwell assay and cell scratch assay indicated that miRNA-155-5p impaired the migration capacity of HUVECs (Figures 3D and 3E). As expected, miRNA-155-5p suppressed tube formation in HUVECs (Figure 3F). Taken together, these results confirmed that miRNA-155-5p impairs endothelial cell functionality and suppresses angiogenesis.

miRNA-155-5p exerts antiangiogenic effects via GDF6-Akt axis

miRNAs exert their biological effects via blocking translation or inducing the degradation of target mRNAs by partial or complete pairing of seed sequence bases with mRNA recognition sites.[14] To identify the target genes of miRNA-155-5p mediating the antiangiogenic effect in HUVECs, a virtual screen was performed using Targetscan and miRanda. As shown in Figure 4A, 11 hits were identified as potential miRNA-155-5p target genes, such as *GDF6*, *Lat2*, *Golph3l*, and *Slc16a2*. KEGG annotation (Figure 4B) and GO enrichment (Figure 4C) analysis of miRNA-155-5p target genes indicated that they were involved in tube morphogenesis and regulation of epithelial cell proliferation. Strikingly, the sequence of miRNA-155-5p in mice and humans are highly similar, and *GDF6* may be one of the target genes involved in the antiangiogenic effect of miRNA-155-5p (Figure 4D). Previous studies reported that angiogenesis is closely related to the *GDF6* gene; GDF6 protein can be recognized by the vascular endothelial growth factor receptor.[15,16] Specifically, human miRNA-155-5p was found to target *GDF6* mRNA 3′ UTR at one binding site of base pair 1994th to 2001th. Subsequently, miRNA-155-5p overexpressed HUVECs were generated by the transfection of miRNA-155-5p agomiR. The abovementioned binding site of *GDF6* mRNA was cloned into a luciferase reporter plasmid and transfected into HEK293T cells. miRNA-155-5p agomiR significantly reduced the luciferase activity of GDF6 reporter, compared to the NC agomiR (Figure 4E), which directly indicated that miRNA-155-5p could partially bind to the 3′ UTR region of *GDF6* mRNA. Furthermore, miRNA-155-5p overexpression led to the suppression of GDF6 protein expression and subsequently impaired Akt phosphorylation (Figure 4F). Thus, miRNA-155-5p suppresses angiogenesis in HUVECs via the GDF6-Akt axis.

miRNA-155-5p delays wound healing in diabetic *db/db* mice

Next, the role of miRNA-155-5p in wound healing was evaluated in diabetic *db/db* mice. Due to its temperature sensitivity and suitable as skin dressing, Pluronic F-127 was chosen as the carrier hydrogel (Figure S2A). The hydrogel-loaded miRNAs did not degrade for up to 10 days (Figure S2B). Full-thickness skin lesions were excised in the interscapular area of each animal, and locally administrated with hydrogel loading NC agomiR, miRNA-155-5p agomiR, NC antagomiR or miRNA-155-5p antagomiR, on days 0 and 3 postwounding (Figure 5A). The expression level of miRNA-155-5p in skin tissues was significantly increased in miRNA-155-5p agomiR-treated mice and decreased in miRNA-155-5p antagomiR-treated mice when compared with their corresponding NC controls (Figure 5B). miRNA-155-5p agomiR or miRNA-155-5p antagomiR treatment did not induce hyperglycemia or body weight changes over 10 days in *db/db* mice (Figures S2C and S2D). For analysis of wound healing, the wounds were monitored every other day, and wound closure rates were calculated for each group.

Figure 2. Sequencing and bioinformatics analysis of exosomal miRNAs

(A) miRNA variety in different groups was analyzed by Venn. (B) PCA of miRNAs from different groups of BMDMs. (C) Representative heatmap of differentially expressed miRNAs in macrophage-derived exosomes. (D) Top 10 highly expressed miRNAs in each group. (E) GO annotations and enrichment analysis of the target genes of miRNAs. (F) KEGG annotations and enrichment analysis of the target genes of miRNAs. (G) Representative volcano map of the differentially expressed miRNAs in M1-Exos and M2-Exos. (H) The expression level of miRNA-155-5p in exosomes from polarized BMDMs. Data are means ± SDs. n = 6. *p < 0.05; **p < 0.01; ***p < 0.001; M1-Exos or M2-Exos versus M0-Exos.

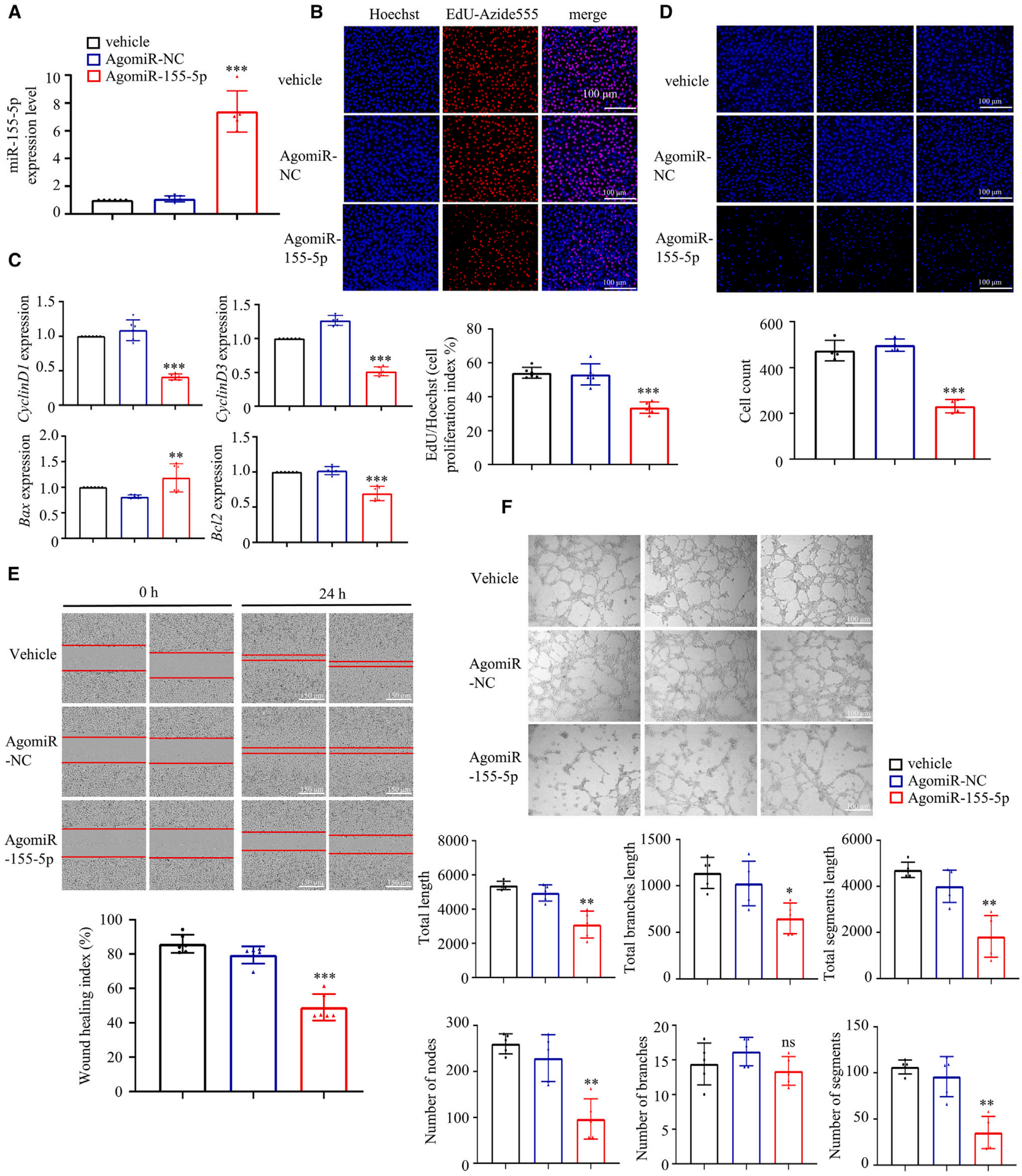

Figure 3. miRNA-155-5p suppresses angiogenesis in HUVECs

(A) Generation of miRNA-155-5p overexpressed HUVECs using agomiR-155-5p. (B) The proliferation of HUVECs assessed by EdU assay. Scale bar, 100 μm. (C) The expression of apoptosis and proliferation-related genes in HUVECs assessed by qRT-PCR. (D) The migration capacity of HUVECs determined by transwell assay. Scale bar, 100 μm. (E) The migration capacity of HUVECs assessed by cell scratch assay. Scale bar, 150 μm. (F) The tube-formation capacity of HUVECs. Scale bar, 100 μm. Data are means ± SDs. n = 6. *p < 0.05; **p < 0.01; ***p < 0.001; AgomiR-NC versus AgomiR-155-5p.

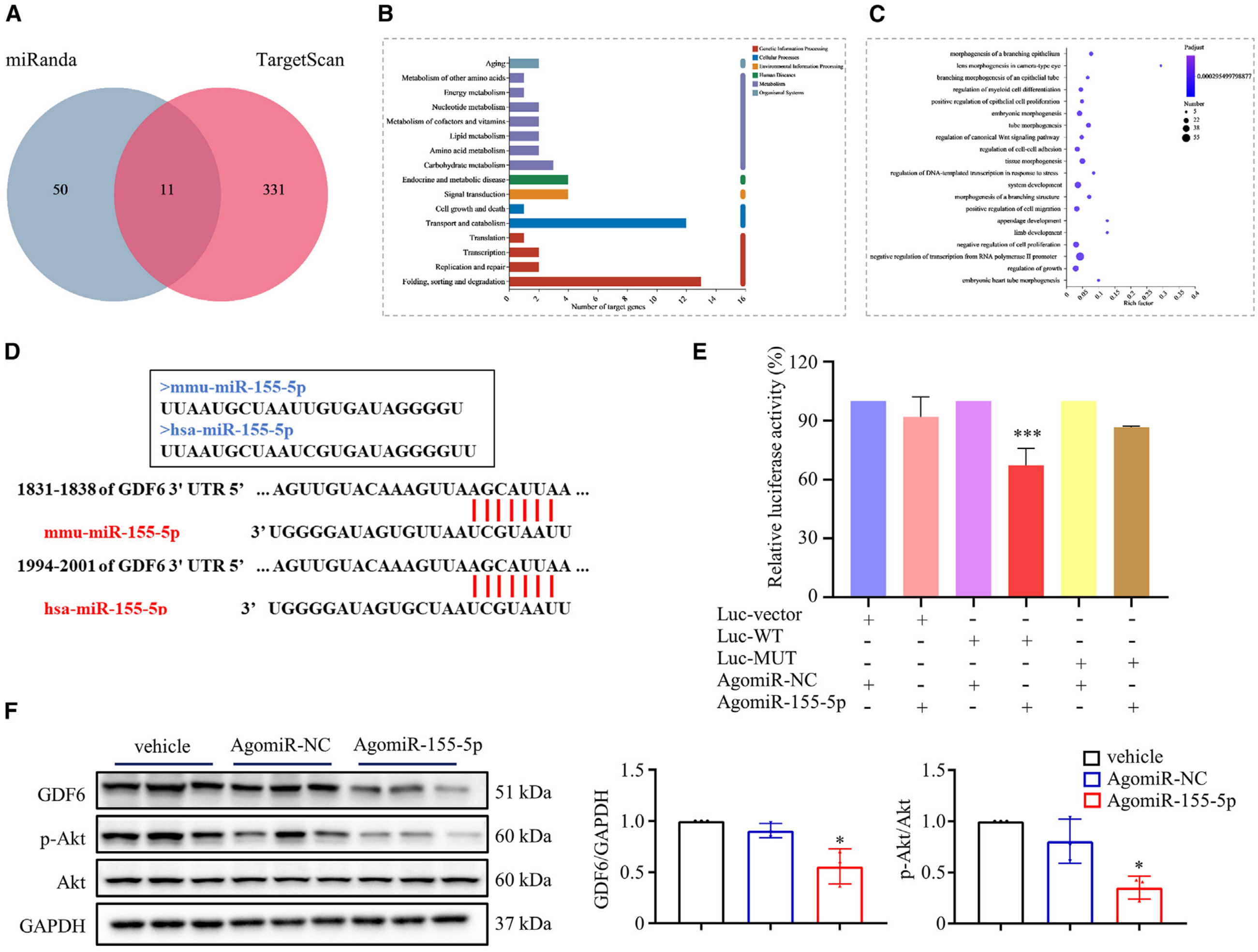

Figure 4. miRNA-155-5p exerts antiangiogenic effects via the GDF6-Akt axis

(A) Identification of 11 genes as miRNA-155-5p target genes using Targetscan and miRanda. (B) KEGG annotations analysis of miRNA-155-5p target genes. (C) GO enrichment analysis of miRNA-155-5p target genes. (D) The binding sites between miRNA-155-5p and *GDF6* mRNA in mice or humans. (E) The effect of agomiR-155-5p on *GDF6* mRNA expression was evaluated with luciferase reporter assay. (F) The protein expression of GDF6, p-Akt, and Akt in HUVECs treated with agomiR-155-5p. GAPDH was used as a loading control. Data are means ± SDs. n = 6. *p < 0.05; ***p < 0.001; AgomiR-NC versus AgomiR-155-5p.

Encouragingly, the miRNA-155-5p antagomiR-treated mice showed 30%, 41%, and 56% closure at 3, 5, and 7 days postinjury and almost completed wound closure by day 9 postinjury, whereas NC antagomiR-treated mice exhibited 20%, 28%, 33%, and 57% closure after 3, 5, 7, and 9 days postinjury, respectively (Figures 5C and 5D). In miRNA-155-5p agomiR-treated mice, the wound closure rates were 13%, 16%, 24%, and 38% at 3, 5, 7, and 9 days postinjury, respectively, and the wound closure rates in the NC agomiR-treated mice were nearly 17%, 26%, 37%, and 57% at 3, 5, 7, and 9 days postinjury, respectively (Figures 5C and 5D). Taken together, these results indicated that miRNA-155-5p antagomiR accelerates wound healing, whereas miRNA-155-5p agomiR impairs wound healing in *db/db* mice.

H&E and Masson's trichrome staining were used to assess the epithelial thickness of regenerated skin. The results showed that the skin epidermis thickness was greater in the miRNA-155-5p antagomiR-treated mice than in the NC antagomiR-treated mice (Figure 6A). The quantitative results revealed that miRNA-155-5p antagomiR-treated mice exhibited the thickest stratum spinosum (Figure S2E). Masson's trichrome staining and Western blotting showed increased collagen deposition in the miRNA-155-5p antagomiR-treated mice compared to the NC antagomiR-treated mice (Figures 6B and S2F). Immunostaining for CD31, a specific marker of vascular vessels, indicated that miRNA-155-5p antagomiR significantly increased microvessel density, whereas miRNA-155-5p agomiR greatly decreased microvessel density, in wounding areas of *db/db* mice, when compared with their corresponding NC controls (Figure 6C). This was further supported with the mRNA expression levels of angiogenic markers, including *SCF*, *SDF-1α*, and *Arg1* (Figure S3). Furthermore, the expression level of GDF6 was increased in the wound area from the miRNA-155-5p

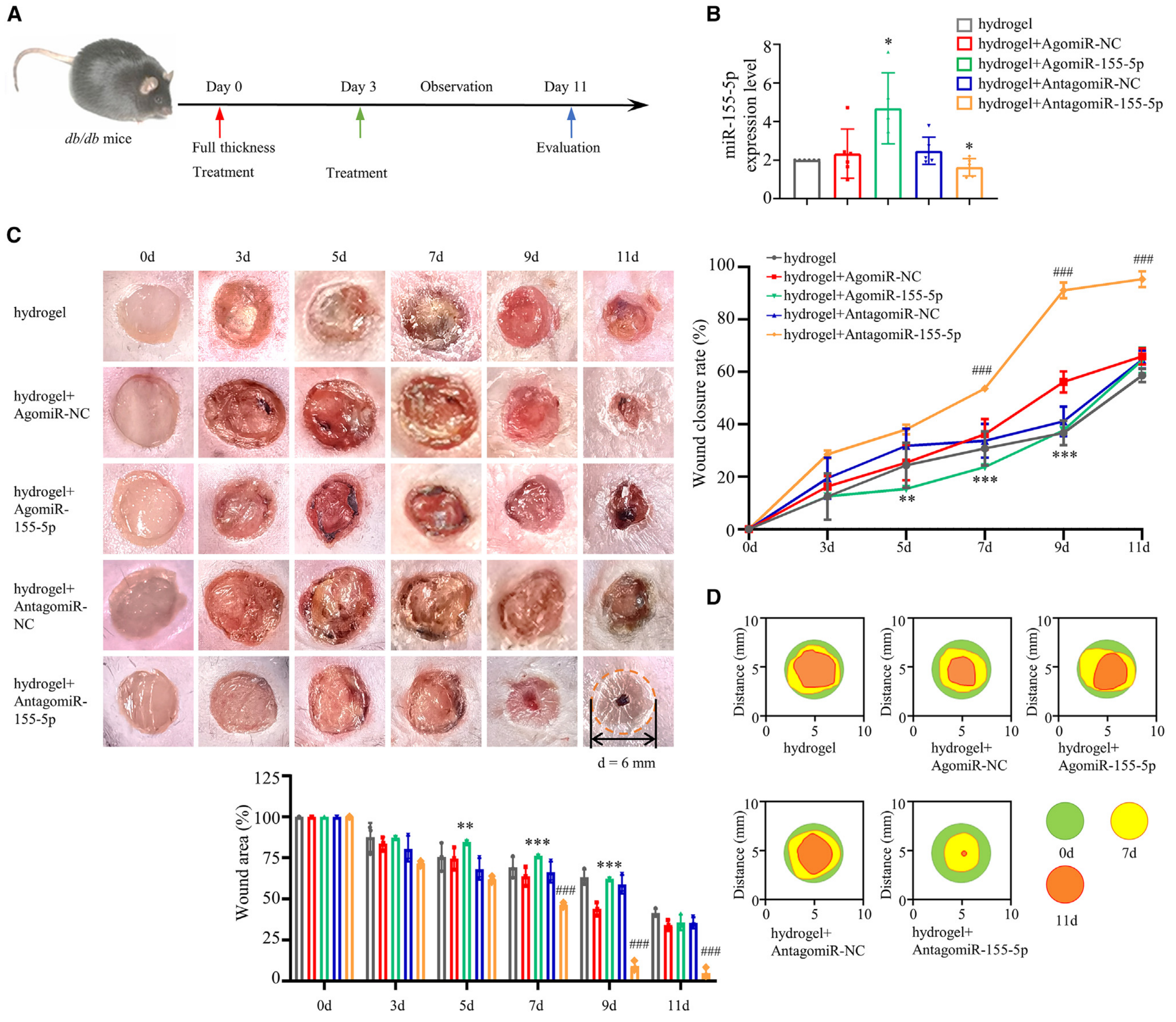

Figure 5. MiRNA-155-5p antagomiR accelerates wound healing in diabetic *db/db* mice

Two full-thickness cutaneous wounds were generated on the backs of *db/db* mice. Animals were randomized into 5 groups and locally treated with 20 μL 25% Pluronic F-127 solution, the same volume of 1.25 mM miRNA-155-5p agomiR in 25% Pluronic F-127 solution, 1.25 mM agomiR NC in 25% Pluronic F-127 solution, 1.25 mM miRNA-155-5p antagomiR in 25% Pluronic F-127 solution, and 1.25 mM antagomiR NC in 25% Pluronic F-127 solution, per wound, on days 0 and 3. n = 6. (A) The experimental procedure of wound healing. (B) The expression levels of miRNA-155-5p in the wound area from different groups of mice. (C) Representative images of wound (left) and wound closure rates of the 5 groups of mice at days 0, 3, 5, 7, 9, and 11 postwounding. (D) Gross view of wounds and area of wounds among the 5 groups of mice. Data are means ± SDs. n = 6. *$p < 0.05$; **$p < 0.01$; ***$p < 0.001$; AgomiR-155-5p versus AgomiR-NC; $^{\#}p < 0.05$; $^{\#\#}p < 0.01$; $^{\#\#\#}p < 0.001$; AntagomiR-155-5p versus AntagomiR-NC.

antagomiR-treated mice compared with the NC antagomiR-treated mice and decreased in the wound area from the miRNA-155-5p agomiR-treated mice compared with the NC agomiR-treated mice (Figure 6E). Taken together, miRNA-155-5p antagomiR accelerates wound healing in diabetic mice by promoting angiogenesis.

DISCUSSION

The unprogrammed macrophage polarization is one of the main physiological features of poor and delayed wound healing.[17] Diabetic wounds show dysregulated and persistent proinflammatory M1-like macrophages, whereas normal wounds display a transition to pro-healing M2-like macrophages at around 3 days postwounding.[4] Several studies have shown that blood glucose affects the wound microenvironment and macrophage polarization.[18] The transition of M1 to M2 macrophages, a dynamic process depending on the surrounding microenvironment, is associated with impaired wound closure, poor angiogenesis, and decreased collagen deposition.[19] It still remains elusive how macrophages orchestrate wound healing.

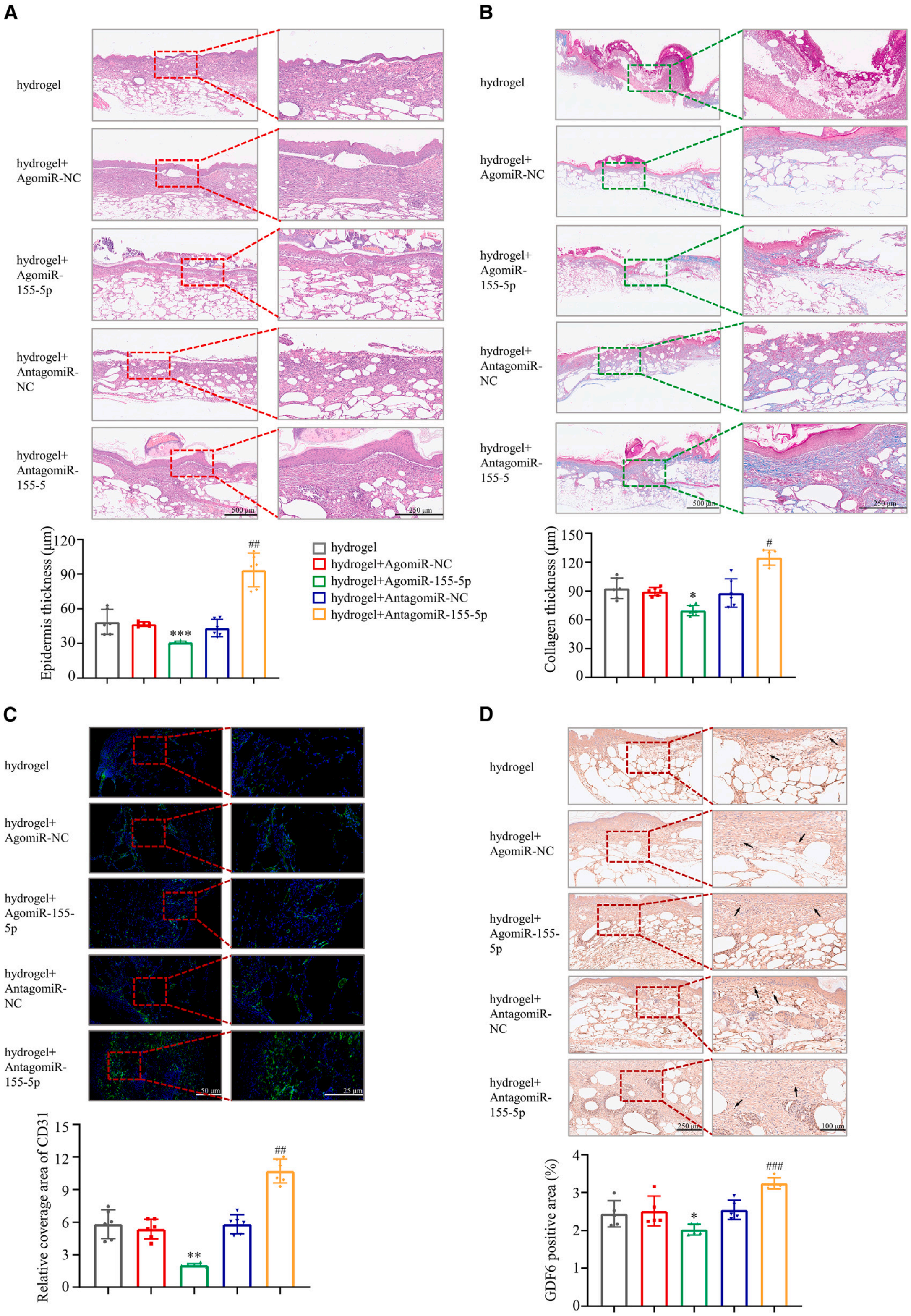

(legend on next page)

After injury, a large number of monocytes and precursor macrophages from bone marrow are recruited around the wound, and these recruited cells exceed the resident macrophage population manifold.[20] Therefore, macrophages derived from BMSCs possess incomparable advantages. Bone marrow monocytes released into the microenvironment express different chemokines and adhesion molecule receptors, and they are preferentially recruited to inflammatory lesions, where they can differentiate into macrophages.[21] In the characterization of activated macrophages, the combination of IFN-γ and tumor necrosis factor signals results in an activated or "inflammatory phenotype" of the macrophage population.[22] It is widely believed that IL-4 rapidly converts resident macrophages into a population of cells programmed to promote wound healing. Meanwhile, IL-4 stimulates arginase activity in macrophages, and promotes collagen and extracellular matrix production in wounding.[23] Thus, BMDMs from nondiabetic mice were recruited to produce exosomes, which were polarized to anti-inflammatory M2-like macrophages and proinflammatory M1-like macrophages via IL-4 and LPS + IFN-γ, respectively. Many studies, either animal or human, have indicated that sustained high glucose exposure reduces macrophage phagocytic activity and renders BMDMs a proinflammatory phenotype.[24] Plasma exosomal miRNAs involved in the adiponectin pathway were significantly dysregulated in diabetic patients.[25] Thus, BMDMs from diabetic mice are more than like M1 macrophages, not M0 macrophages, which could not satisfy our current research purpose. Isolation of BMDMs from diabetic mice and evaluation of exosomal miRNA expression profiles from them could be interesting. It could help to identify miRNAs differently expressed in BMDMs from diabetic mice and develop them as therapeutic agents against diabetes.

Emerging evidence indicates that exosomes are involved in wound healing through multiple pathways, including angiogenesis and collagen synthesis. Exosomes derived from adipose stem cells accelerate diabetic wound healing by promoting angiogenesis and collagen synthesis.[26] Fibroblast-derived exosomes induce keratinocyte migration and proliferation *in vitro* and accelerate wound healing in diabetic mice.[27] In addition, macrophage-derived exosomes accelerate wound healing in diabetic rats by reducing the secretion of proinflammatory cytokines.[28] Moreover, exosomes derived from other cells modulate diabetic wound healing by inducing or suppressing macrophage polarization in an indirect manner.[27,29] In a recent study, M2 macrophage-derived exosomes promote M1 to M2 macrophage transform in the wound site, which accelerates wound healing by enhancing angiogenesis and epithelial regeneration.[8] Our study showed that the exosomes from polarized BMDMs mediate cellular communication between macrophages and endothelial cells. Thus, exosomes regulate the functions of macrophages and endothelial cells in various stages of wound healing, which could be a promising therapeutic strategy against DFUs.

As critical posttranscriptional regulators, miRNAs participate in many physiological and pathological processes. One miRNA can simultaneously target multiple genes, forming a network amplification effect.[30] miRNA is relatively stable and feasible for clinical application. Several studies have reported the critical roles of miRNAs in wound healing. The inhibition of miRNA-26a in diabetic wounds promotes the formation of granulation tissue and blood vessels.[31] miRNA-27b promotes proliferation, migration, and tube formation of bone marrow–derived angiogenic cells to accelerate wound healing in diabetic mice.[32] The inhibition of miRNA-29a increases collagen content and corrects the impaired biomechanical properties of diabetic skin.[33] Our study deciphered the miRNA profiles in exosomes derived from polarized BMDMs. A series of differently expressed miRNAs were identified. Among them, miRNA-155-5p was found to be highly expressed in M1-Exos. The overexpression of miRNA-155-5p impairs the migration and angiogenesis of HUVECs, and the inhibition of miRNA-155-5p promotes wound healing in diabetic mice. Dysregulation of miRNA-155 expression was found to affect the development of diabetic nephropathy and retinopathy.[34] Thus, miRNAs are considered to be attractive candidates for the treatment of DFUs.

In animals, miRNAs bind to specific sequences in the 3′ UTR, causing degradation or translation arrest of the target mRNAs. GDF6 was predicted to be one of the targets of miRNA-155-5p. GDF6, a member of the superfamily transforming growth factor-β, induces vascular smooth muscle cell growth and remodeling to promote endothelial vascular integrity.[15,16] GDF6 treatment forms new tendons and promotes tendon regeneration in bone marrow mesenchymal stem cells.[35] High-temperature requirement factor A1 regulates angiogenesis by GDF6 in the retinas of mice.[36] In addition, another predicted target of miRNA-155-5p was fibroblast growth factor 7 (FGF7). We found that the protein level of FGF7 was decreased in miRNA-155-5p overexpressed HUVECs (Figure S4). FGF7 participated in various processes such as angiogenesis and wound healing.[37] A previous study suggested that miRNA-155 inhibition restores FGF7 expression in diabetic skin and decreases wound inflammation.[38] Thus, miRNA-155-5p could target multiple mRNAs, including GDF6 and FGF7 to regulate angiogenesis and tissue remodeling.

Systemic delivery, typically oral or intravenous, is the most common method of drug administration. Unfortunately, systemic drug delivery relies on the adequate perfusion of target tissue, and many chronic wounds lack this critical blood supply.[39] Owing to the risk

Figure 6. Hydrogel loaded-antagomiR-155-5p promotes collagen deposition and angiogenesis in *db/db* mice

(A) H&E staining of wound area and epidermis thickness in different groups of mice. Scale bar: left, 500 μm; right, 250 μm. (B) Masson trichrome staining of wound area and collagen accumulation in different groups of mice. Scale bar: left, 500 μm; right, 250 μm. (C) CD31 and DAPI double staining in wound bed of mice after 11 days postinjury. Scale bar: left, 50 μm; right, 25 μm. (D) Immunohistochemical staining of GDF6 in wound area from different groups of mice. The quantitative analysis of GDF6 was performed according to the IRS. Scale bar: left, 250 μm; right, 100 μm. Data are means ± SDs. n = 6. *p < 0.05; **p < 0.01; ***p < 0.001; AgomiR-155-5p versus AgomiR-NC; #p < 0.05; ##p < 0.01; ###p < 0.001; AntagomiR-155-5p versus AntagomiR-NC.

of multiorgan toxicity and the unpredictability of drug delivery to target tissue, scientists are increasingly turning their research to the local delivery of drugs to promote wound healing. Local drug delivery can deliver the bulk drug substance to the target area, thereby maximizing the therapeutic potential and reducing drug toxicity in other organs.[40] With the development of therapeutic nucleic acids, there are many ways to deliver nucleic acid drugs for wound healing. Exosomes carrying special nucleic acids have been collected for wound healing.[41] Unfortunately, exosome-mediated nucleic acids delivery still need to overcome some challenges for clinical translations, such as insufficient active ingredients and low exosome activity.[42] The use of protective substrates such as hydrogel represents one way to overcome these challenges.[39,43] Accumulating evidence has indicated that Pluronic F-127, a thermosensitive hydrogel, could serve as a promising vehicle for miRNA encapsulation. The release of miRNA-148b mimics from Pluronic F-127 hydrogel enhances endothelial cell function, thereby increasing angiogenesis.[44] The treatment of miRNA-129-2-3p mimics in Pluronic F-127 hydrogel accelerates wound healing.[45] In the present study, miRNA-155-5p agomiR- and antagomiR-loaded Pluronic F-127 hydrogels were prepared, which was a safe and effective method for the local delivery of miRNAs.

Compared with miRNA mimics and inhibitors, agomiR for miRNA overexpression and antagomiR for miRNA suppression are more suitable for *in vivo* interference experiments. They exhibit higher stability and inhibitory effect in animal studies. In addition, miRNA agomiR and antagomiR are double-stranded, which can be directly loaded into RNA-induced silencing complexes and function immediately without Dicer enzyme processing. Instead of delivering the miRNA-155-5p-related products by conventional PBS injection, we creatively used temperature-sensitive hydrogel as carrier and dressing. miRNA was incorporated into thermosensitive hydrogels to form nucleic acid hybrid hydrogels, which showed the advantages of sustained release and practicability.[46] The hydrogel loaded with miRNA-155-5p antagomiR was locally administrated, which promoted collagen accumulation and reepithelialization at the wound site, and significantly accelerated wound healing in diabetic mice.

In conclusion, our study deciphered the miRNA expression profiles in exosomes derived from polarized BMDMs, and identified that miRNA-155-5p, highly expressed in M1-Exos, suppresses endothelial cell functions and angiogenesis. miRNA-155-5p antagomiR could be a promising nucleic acid therapy against DFUs.

MATERIALS AND METHODS

Isolation of BMDMs

BMDMs were prepared from 6- to 8-week-old male C57BL/6J mice. Bone marrow was isolated from the femurs and tibias using a 5-mL syringe and was cultured in DMEM (Gibco, Carlsbad, CA) with 10% fetal bovine serum (FBS, Gibco), 1% penicillin-streptomycin (Gibco), and 10% L929 cell supernatant for 7 days. BMDMs were treated with 20 ng/mL IFN-γ (Peprotech, Cranbury, NJ) and 100 ng/mL LPS (Sigma-Aldrich, St. Louis, MO) for 1 day to differentiate into M1-like macrophages. Alternatively, BMDMs were treated with 40 ng/mL IL-4 (Peprotech) for 1 day to differentiate into M2-like macrophages. All of the cells were cultured in a humidified incubator containing 5% CO_2 at 37°C.

Isolation and characterization of exosomes

BMDMs were incubated for 2 days in serum-free DMEM. Subsequently, the cell culture supernatant was collected and centrifuged at 1,000 $\times$ *g* for 30 min at 4°C to remove cell debris. Total exosome isolation reagent (Thermo Fisher Scientific, Carlsbad, CA) was added to the cell culture medium (1:2). After incubation overnight at 4°C, the mixture was centrifuged at 10,000 $\times$ *g* for 1 h. After removing the supernatant, the precipitate was resuspended in PBS. Exosome size distribution was determined with dynamic light scattering (Zetasizer Nano, Malvern, UK). For TEM analysis, 10 μL exosome solution was dropped onto the copper net. The copper mesh was stained with 3% uranyl acetate for 2 min. After drying for 10 min, the copper mesh was observed using a JEM-1200EX TEM (JEOL, Tokyo, Japan).

Labeling and cellular uptake of exosomes

Exosomes were labeled with a membrane-labeling dye, DiI (Thermo Fisher Scientific), according to the manufacturer's protocol. Exosomes were incubated in a DiI staining solution (5 μg/mL) for 10 min at room temperature. After being washed with PBS, the exosomes were centrifuged at 10,000 $\times$ *g* for 5 min. HUVECs were treated with the labeled exosomes for 3 h at 37°C. Cell nuclei were stained with DAPI (Sigma-Aldrich), and microfilaments were stained with Actin-Tracker Green-488 (Beyotime, Shanghai, China). The fluorescent images were captured by a confocal fluorescence microscope (Leica TCS SP8, Leica, Weztlar, Germany).

Cell culture and transfection with miRNA

HUVECs were obtained from the American Type Culture Collection (Rockville, MD). Cells were cultured in complete endothelial cell medium (ECM) supplemented with 5% FBS, 1% endothelial cell growth supplement, and 1% penicillin-streptomycin in a humidified atmosphere containing 5% CO_2 at 37°C. For transfection, HUVECs were seeded in 6-well plates and cultured overnight. A total of 100 nM miRNA agomiR or NC (IGE, Guangzhou, China) were transfected into HUVECs using Lipofectamine 3000 reagent (Invitrogen, Waltham, MA). Cells at passages between two and five were used in all of the experiments.

CCK-8 assay

CCK-8 was used to determine cell viability. HUVECs were seeded into 96-well plates at a density of 2 $\times$ 10^3 cells per well. Cells were treated with exosomes for 6 h. Subsequently, 10 μL CCK-8 solution (Beyotime) was added to each well, followed by incubation for 1 h at 37 °C. Absorbance at 450 nm was measured using a microplate reader (FlexStation 3 Multi Mode, San Jose, CA).

EdU assay

HUVECs were seeded in 6-well plates. EdU staining was conducted using BeyoClick EdU Cell Proliferation Kit (with Alexa Fluor 555,

Beyotime) according to the manufacturer's instruction. Cell nuclei were stained with Hoechst (Beyotime). The images were captured by the Leica DMi8 microscope (Leica).

Cell scratch assay

HUVECs were seeded into 6-well plates at a density of 6×10^5 cells per well. A single scratch was made using a sterile 200-μL pipette tip. The nonadherent cells were removed by rinsing with PBS three times. Then, the cells were incubated with serum-free ECM for 24 h. Images of the scratches were captured with IncuCyte real-time dynamic cell imaging analysis system (Essen Bioscience, Ann Arbor, MI) at 10× magnification. The areas of the scratches were analyzed using ImageJ (NIH, Bethesda, MD).

Transwell migration assay

Transwell migration assay was performed using 24-well transwell plates and inserts with a filter of 8 μm pore (Corning, Glendale, AZ). HUVECs were seeded into the upper chamber of the insert at a concentration of 1.5×10^5 cells in serum-free ECM, and complete ECM was added to the lower chamber. After incubation for 6 h, the cells on the top surface of the membrane were wiped off, and the cells on the lower surface were fixed with 4% paraformaldehyde. Then, the fixed cells were stained with DAPI. Fluorescent images were captured with the Leica DMi8 microscope (Leica) at 10× magnification. The cell numbers were counted with ImageJ.

Tube formation assay

Matrigel (BD Biosciences, Glendale, AZ) was added to 96-well plates at 50 μL per well. HUVECs was seeded to each well at a concentration of 4×10^4 in ECM (50 μL). HUVECs were treated with exosomes or miRNAs and then incubated for 5 h at 37°C. The tube formations were observed using IncuCyte real-time dynamic cell imaging analysis system at 4× magnification. The results were analyzed by ImageJ.

qRT-PCR

Total RNAs were extracted using Trizol reagent (Invitrogen) according to a previous publication.[47] The cDNA was synthesized from 1 μg RNA using the TaqMan MicroRNA Reverse Transcription Kit (Applied Biosystems, Waltham, MA) or the SuperScript III First-Strand Synthesis System (Invitrogen) following the manufacturer's instructions. The qPCR experiments were conducted on the Step-One plus real-time PCR System using SYBR Green PCR Master Mix (Invitrogen) with gene-specific primers (Table S2). The qRT-PCR assay was biologically repeated at least three times. *β-Actin* was applied as the internal standard. For miRNA expression, Ct values were normalized to U6 as the reference gene. The expression levels of genes were finally quantitated using the standard $2^{-\Delta\Delta Ct}$ method.

Western blotting

HUVECs and mouse skins were lysed with radioimmunoprecipitation assay lysis buffer (Beyotime) as described previously.[48] Protein concentration was determined using a BCA Protein Assay Kit (Thermo Fisher). Equal amounts of proteins (20–30 μg) were separated via 10% SDS-PAGE, transferred to polyvinylidene fluoride membranes, blocked with 5% fat-free milk for 1 h at room temperature, and subsequently incubated with primary antibodies overnight at 4°C. The membranes were washed with Tris-buffered saline, 0.1% Tween 20 3 times, 30 min each, and incubated in a blocking solution with horseradish peroxidase–tagged second antibodies for 1.5 h at room temperature. Signals were developed using a SuperSignal West Femto Maximum Sensitivity Substrate kit (Thermo Fisher). Subsequently, specific protein bands were visualized using the ChemiDoc MP Imaging System, and quantification was performed with Image Lab 5.1 (Bio-Rad, Hercules, CA). The antibodies used in this study included Alix (Abcam [Cambridge, UK], ab117600, 1:1,000), TSG101 (Abcam, ab30871, 1:1,000), GDF6 (Abcam, ab226853, 1:1,000), FGF7 (Abcam, ab131162, 1:1,200), phospho-Akt (Ser473) (Cell Signaling Technology [Danvers, MA], catalog no. 4060, 1:1,000), Akt (Cell Signaling Technology, catalog no. 4691, 1:1,000), Collagen I (Abcam, ab260043, 1:1,000), and glyceraldehyde 3-phosphate dehydrogenase (GAPDH) (Cohesion Biosciences [London, UK], CPA9295, 1:5,000).

miRNA library construction and sequencing

Total RNAs from exosomes were used for miRNA library preparation and sequencing by the Majorbio group (Shanghai, China). Briefly, total RNA samples were fractionated on a 15% Tris-borate-EDTA polyacrylamide gel (Invitrogen), and sRNAs ranging between 18 and 30 nt were used for library preparation. sRNAs were reverse transcribed and amplified by PCR. The PCR products were sequenced using the Illumina HiSeq 2500 platform. The RNA sequencing raw data were deposited in GEO: GSE234953.

Bioinformatics analysis

The Quantifier script in the miRDeep2 software was used to analyze the miRNA expression. Venn correlation and PCA analysis were performed according to the expression of miRNA among different samples. Three software programs, DESeq2, DEGseq, and edgeR, were used to analyze the differences in miRNA expression. The false discovery rate correction with Benjamini-Hochberg method was used to correct the statistical test results of miRNA expression differences. The predicted miRNA target genes were compared with six major databases (GO, KEGG, Clusters of Orthologous Genes [COG], NR, SwisS-PROT, and Pfam) to obtain comprehensive functional annotation information about the target genes. The target genes were sequentially aligned with the NR, SwisS-ProT, and COG databases using DIAMOND software. BLAST2GO was used for sequence alignment with the GO database. KEGG Orthology results were obtained using KOBAS2.1.

Dual-luciferase reporter assay

HEK293T cells were cotransfected with the GDF6 promoter luciferase reporter plasmid and the hsa-miRNA-155-5p agomiR (Genepharma, Shanghai, China) in the presence of an empty vector. The luciferase activities were assessed using the Dual-Luciferase Reporter Assay System (Promega, Madison, WI) according to the manufacturer's protocol. The results were standardized with the activity of Renilla luciferase.

Wound healing in *db/db* mice

All of the animal experiments were approved by the Animal Ethical and Welfare Committee of the University of Macau (no. UMARE-033-2017). All of the procedures involved in the animal experiments were carried out in accordance with the approved guidelines and regulations. Male *Lepr*^{em2Cd479}*/Gpt* diabetic mice (*db/db*) were purchased from Gempharmatech (Nanjing, China) and housed in the animal facility of the University of Macau, maintained at 23°C ± 1°C (50% ± 5% relative humidity) with 12-h light/dark cycles with free access to water and a regular chow diet.

miRNA-155-5p agomiR, agomiR negative control (agomer NC), miRNA-155-5p antagomiR, and antagomiR NC were purchased from Genepharma. Pluronic F-127 (Sigma-Aldrich) hydrogel powder was mixed with diethyl pyrocarbonate water to obtain a 25% (w/v) solution. All of the procedures were carried out under aseptic and nonenzymatic conditions. *db/db* mice (10–12 weeks old, fasting blood glucose >11.1 mmol/L) were randomly divided into 5 groups of 6 mice each. The 5 groups of *db/db* mice were locally administrated with 20 μL 25% Pluronic F-127 solution, the same volume of 1.25 mM miRNA-155-5p agomiR in 25% Pluronic F-127 solution, 1.25 mM agomiR NC in 25% Pluronic F-127 solution, 1.25 mM miRNA-155-5p antagomiR in 25% Pluronic F-127 solution, and 1.25 mM antagomiR NC in 25% Pluronic F-127 solution, respectively, per wound. The administration was performed on days 0 and 3 of the wound closure experiment.

Assessment of wound area closure

Mice were anesthetized via inhalation of 3% isoflurane (RWD, Shenzhen, China). Before excision for wounds, the dorsal hair was shaved with an electric clipper followed by a depilatory cream. The skin was rinsed with alcohol, and two full-thickness wounds extending through the panniculus carnosus were created on the dorsum on each side of midline, using a 6-mm biopsy punch. Digital photographs were recorded on the day of surgery and every other day post-injury at fixed focal length and distance. Changes of wounds were quantified by assessing the wound area on days 0, 3, 5, 7, 9, and 11 using ImageJ software. The wound closure rates were calculated as the following formulation: (wound area on day 0 − wound area on day X)/wound area on day 0 × 100%. On day 11, the mice were euthanized, and wound tissues were dissected. Part of the wound tissues was fixed in 4% paraformaldehyde at 4°C overnight and then embedded in paraffin. The remaining tissues were frozen in liquid nitrogen and stored in −80°C.

Histological analysis and microvessel density assay

For histological evaluation, sections (5 μm) were deparaffinized and rehydrated, followed by H&E and Masson's trichrome staining, according to standard protocols as described previously.[49] Epidermal thickness was measured on images of H&E staining slides taken at 20× magnification. Collagen quantification was determined on Masson trichrome–stained skin areas using ImageJ software. For immunohistochemical staining of CD31, the wound tissue sections were deparaffinized and stained with CD31 antibody (Abcam, ab182981, 1:800). Five randomly high-power field areas with the highest microvessel density were selected for each section. The average was calculated as the microvessel density.

Immunohistochemistry

Paraffin-embedded sections of skin tissues were stained using the avidin-biotin complex. Sections were incubated with polyclonal rabbit anti-GDF6 antibody at 4°C overnight, followed by treatment with horseradish peroxidase–labeled anti-rabbit immunoglobulin G. The streptavidin-biotin complex was used to visualize the staining. For each section, five high-power fields were observed. Scoring was conducted according to the immunoreactive score (IRS) standard.[46]

Statistical analysis

Data were analyzed using GraphPad Prism version 8.3.0 (GraphPad Software, San Diego, CA). All of the experimental data were expressed as mean ± SD, and the sample size for each experiment corresponds to three biological replicates. Two groups were compared via the Student's t test, and more than two groups were compared using one-way ANOVAs, considering $p < 0.05$ as significant differences. Where statistical significance is evaluated, variance between groups is confirmed to be similar between comparison groups (control versus experimental) and the statistical analysis is deemed appropriate.

DATA AND CODE AVAILABILITY

The authors confirm that the data supporting the findings of this study are available within the article and its supplemental information. The RNA sequencing raw data have been deposited in GEO: GSE234953. Raw data are available from the corresponding author upon reasonable request.

SUPPLEMENTAL INFORMATION

Supplemental information can be found online at https://doi.org/10.1016/j.omtn.2023.102074.

ACKNOWLEDGMENTS

This work was supported by the National Natural Science Foundation of China (82073715); the Science and Technology Development Fund, Macau SAR (file no. FDCT 0001/2021/AKP, SKL-QRCM(UM)-2023-2025), and the Research Fund of University of Macau (MYRG2020-00091-ICMS, MYRG2022-00177-ICMS).

AUTHOR CONTRIBUTIONS

Conceptualization: L.L. Investigation: R.L. and T.Z. Methodology: R.L., T.Z., X.C., C.W., B.G., and L.L. Data curation: J.C., F.Z., and L.L. Resources: T.Z., X.C., C.W., and B.G. Writing – original draft: R.L. Writing – review & editing: L.L. Funding acquisition: L.L. Project administration: L.L. Supervision: L.L.

DECLARATION OF INTERESTS

The authors declare no competing interests.

REFERENCES

1. Basu Mallik, S., Jayashree, B.S., and Shenoy, R.R. (2018). Epigenetic modulation of macrophage polarization- perspectives in diabetic wounds. J. Diabet. Complicat. *32*, 524–530.
2. Armstrong, D.G., Boulton, A.J.M., and Bus, S.A. (2017). Diabetic foot ulcers and their recurrence. N. Engl. J. Med. *376*, 2367–2375.
3. Hesketh, M., Sahin, K.B., West, Z.E., and Murray, R.Z. (2017). Macrophage phenotypes regulate scar formation and chronic wound healing. Int. J. Mol. Sci. *18*, 1545.
4. Louiselle, A.E., Niemiec, S.M., Zgheib, C., and Liechty, K.W. (2021). Macrophage polarization and diabetic wound healing. Transl. Res. *236*, 109–116.
5. Sindrilaru, A., Peters, T., Wieschalka, S., Baican, C., Baican, A., Peter, H., Hainzl, A., Schatz, S., Qi, Y., Schlecht, A., et al. (2011). An unrestrained proinflammatory M1 macrophage population induced by iron impairs wound healing in humans and mice. J. Clin. Invest. *121*, 985–997.
6. Tkach, M., and Théry, C. (2016). Communication by extracellular vesicles: Where we are and where we need to go. Cell *164*, 1226–1232.
7. Whitham, M., Parker, B.L., Friedrichsen, M., Hingst, J.R., Hjorth, M., Hughes, W.E., Egan, C.L., Cron, L., Watt, K.I., Kuchel, R.P., et al. (2018). Extracellular vesicles provide a means for tissue crosstalk during exercise. Cell Metabol. *27*, 237–251.e4.
8. Kim, H., Wang, S.Y., Kwak, G., Yang, Y., Kwon, I.C., and Kim, S.H. (2019). Exosome-guided phenotypic switch of M1 to M2 macrophages for cutaneous wound healing. Adv. Sci. *6*, 1900513.
9. Bartel, D.P. (2004). MicroRNAs: genomics, biogenesis, mechanism, and function. Cell *116*, 281–297.
10. Lou, R., Chen, J., Zhou, F., Wang, C., Leung, C.H., and Lin, L. (2022). Exosome-cargoed microRNAs: Potential therapeutic molecules for diabetic wound healing. Drug Discov. Today *27*, 103323.
11. Li, D., Zhang, T., Lu, J., Peng, C., and Lin, L. (2020). Natural constituents from food sources as therapeutic agents for obesity and metabolic diseases targeting adipose tissue inflammation. Crit. Rev. Food Sci. Nutr. *61*, 1947–1965.
12. Lavin, Y., Mortha, A., Rahman, A., and Merad, M. (2015). Regulation of macrophage development and function in peripheral tissues. Nat. Rev. Immunol. *15*, 731–744.
13. Zheng, Q., and Wang, X.J. (2008). GOEAST: a web-based software toolkit for Gene Ontology enrichment analysis. Nucleic Acids Res. *36*, W358–W363.
14. Agarwal, V., Bell, G.W., Nam, J.W., and Bartel, D.P. (2015). Predicting effective microRNA target sites in mammalian mRNAs. Elife *4*, 05005.
15. Wu, R., Hu, W., Chen, H., Wang, Y., Li, Q., Xiao, C., Fan, L., Zhong, Z., Chen, X., Lv, K., et al. (2021). A novel human long noncoding RNA SCDAL promotes angiogenesis through SNF5-mediated GDF6 expression. Adv. Sci. *8*, e2004629.
16. Krispin, S., Stratman, A.N., Melick, C.H., Stan, R.V., Malinverno, M., Gleklen, J., Castranova, D., Dejana, E., and Weinstein, B.M. (2018). Growth differentiation factor 6 promotes vascular stability by restraining vascular endothelial growth factor signaling. Arterioscler. Thromb. Vasc. Biol. *38*, 353–362.
17. Ayala, T.S., Tessaro, F.H.G., Jannuzzi, G.P., Bella, L.M., Ferreira, K.S., and Martins, J.O. (2019). High glucose environments interfere with bone marrow-derived macrophage inflammatory mediator release, the TLR4 pathway and glucose metabolism. Sci. Rep. *9*, 11447.
18. Huang, S.M., Wu, C.S., Chiu, M.H., Wu, C.H., Chang, Y.T., Chen, G.S., and Lan, C.C. (2019). High glucose environment induces M1 macrophage polarization that impairs keratinocyte migration via TNF-α: An important mechanism to delay the diabetic wound healing. J. Dermatol. Sci. *96*, 159−167.
19. Ganesh, G.V., and Ramkumar, K.M. (2020). Macrophage mediation in normal and diabetic wound healing responses. Inflamm. Res. *69*, 347–363.
20. Davies, L.C., Jenkins, S.J., Allen, J.E., and Taylor, P.R. (2013). Tissue-resident macrophages. Nat. Immunol. *14*, 986–995.
21. Gordon, S., and Taylor, P.R. (2005). Monocyte and macrophage heterogeneity. Nat. Rev. Immunol. *5*, 953–964.
22. O'Shea, J.J., and Murray, P.J. (2008). Cytokine signaling modules in inflammatory responses. Immunity *28*, 477–487.
23. Kreider, T., Anthony, R.M., Urban, J.F., Jr., and Gause, W.C. (2007). Alternatively activated macrophages in helminth infections. Curr. Opin. Immunol. *19*, 448–453.
24. Pavlou, S., Lindsay, J., Ingram, R., Xu, H., and Chen, M. (2018). Sustained high glucose exposure sensitizes macrophage responses to cytokine stimuli but reduces their phagocytic activity. BMC Immunol. *19*, 24.
25. Santovito, D., De Nardis, V., Marcantonio, P., Mandolini, C., Paganelli, C., Vitale, E., Buttitta, F., Bucci, M., Mezzetti, A., Consoli, A., and Cipollone, F. (2014). Plasma exosome microRNA profiling unravels a new potential modulator of adiponectin pathway in diabetes: effect of glycemic control. J. Clin. Endocrinol. Metab. *99*, E1681–E1685.
26. Hyldig, K., Riis, S., Pennisi, C.P., Zachar, V., and Fink, T. (2017). Implications of extracellular matrix production by adipose tissue-derived stem cells for development of wound healing therapies. Int. J. Mol. Sci. *18*, 1167.
27. Liu, W., Yu, M., Xie, D., Wang, L., Ye, C., Zhu, Q., Liu, F., and Yang, L. (2020). Melatonin-stimulated MSC-derived exosomes improve diabetic wound healing through regulating macrophage M1 and M2 polarization by targeting the PTEN/AKT pathway. Stem Cell Res. Ther. *11*, 259.
28. Li, M., Wang, T., Tian, H., Wei, G., Zhao, L., and Shi, Y. (2019). Macrophage-derived exosomes accelerate wound healing through their anti-inflammation effects in a diabetic rat model. Artif. Cells, Nanomed. Biotechnol. *47*, 3793–3803.
29. Shi, R., Jin, Y., Zhao, S., Yuan, H., Shi, J., and Zhao, H. (2022). Hypoxic ADSC-derived exosomes enhance wound healing in diabetic mice via delivery of circ-Snhg11 and induction of M2-like macrophage polarization. Biomed. Pharmacother. *153*, 113463.
30. O'Brien, J., Hayder, H., Zayed, Y., and Peng, C. (2018). Overview of microRNA biogenesis, mechanisms of actions, and circulation. Front. Endocrinol. *9*, 402.
31. Icli, B., Wara, A.K.M., Moslehi, J., Sun, X., Plovie, E., Cahill, M., Marchini, J.F., Schissler, A., Padera, R.F., Shi, J., et al. (2013). MicroRNA-26a regulates pathological and physiological angiogenesis by targeting BMP/SMAD1 signaling. Circ. Res. *113*, 1231–1241.
32. Wang, J.M., Tao, J., Chen, D.D., Cai, J.J., Irani, K., Wang, Q., Yuan, H., and Chen, A.F. (2014). MicroRNA miR-27b rescues bone marrow-derived angiogenic cell function and accelerates wound healing in type 2 diabetes mellitus. Arterioscler. Thromb. Vasc. Biol. *34*, 99–109.
33. Zgheib, C., Hodges, M., Hu, J., Beason, D.P., Soslowsky, L.J., Liechty, K.W., and Xu, J. (2016). Mechanisms of mesenchymal stem cell correction of the impaired biomechanical properties of diabetic skin: The role of miR-29a. Wound Repair Regen. *24*, 237–246.
34. Jankauskas, S.S., Gambardella, J., Sardu, C., Lombardi, A., and Santulli, G. (2021). Functional role of miR-155 in the pathogenesis of diabetes mellitus and its complications. Noncoding. RNA *7*, 7030039.
35. Chai, W., Ni, M., Rui, Y.F., Zhang, K.Y., Zhang, Q., Xu, L.L., Chan, K.M., Li, G., and Wang, Y. (2013). Effect of growth and differentiation factor 6 on the tenogenic differentiation of bone marrow-derived mesenchymal stem cells. Chin. Med. J. *126*, 1509–1516.
36. Zhang, L., Lim, S.L., Du, H., Zhang, M., Kozak, I., Hannum, G., Wang, X., Ouyang, H., Hughes, G., Zhao, L., et al. (2012). High temperature requirement factor A1 (HTRA1) gene regulates angiogenesis through transforming growth factor-β family member growth differentiation factor 6. J. Biol. Chem. *287*, 1520–1526.
37. Liu, Y., Liu, Y., Deng, J., Li, W., and Nie, X. (2021). Fibroblast growth factor in diabetic foot ulcer: Progress and therapeutic prospects. Front. Endocrinol. *12*, 744868.
38. Moura, J., Sørensen, A., Leal, E.C., Svendsen, R., Carvalho, L., Willemoes, R.J., Jørgensen, P.T., Jenssen, H., Wengel, J., Dalgaard, L.T., and Carvalho, E. (2019). microRNA-155 inhibition restores Fibroblast Growth Factor 7 expression in diabetic skin and decreases wound inflammation. Sci. Rep. *9*, 5836.
39. Whittam, A.J., Maan, Z.N., Duscher, D., Wong, V.W., Barrera, J.A., Januszyk, M., and Gurtner, G.C. (2016). Challenges and opportunities in drug delivery for wound healing. Adv. Wound Care *5*, 79–88.
40. Jo, J.I., Gao, J.Q., and Tabata, Y. (2019). Biomaterial-based delivery systems of nucleic acid for regenerative research and regenerative therapy. Regen. Ther. *11*, 123–130.
41. Xu, J., Bai, S., Cao, Y., Liu, L., Fang, Y., Du, J., Luo, L., Chen, M., Shen, B., and Zhang, Q. (2020). miRNA-221-3p in endothelial progenitor cell-derived exosomes accelerates skin wound healing in diabetic mice. Diabetes Metab. Syndr. Obes. *13*, 1259–1270.

42. Shen, J., Zhao, X., Zhong, Y., Yang, P., Gao, P., Wu, X., Wang, X., and An, W. (2022). Exosomal ncRNAs: The pivotal players in diabetic wound healing. Front. Immunol. *13*, 1005307.

43. Doi, N., Jo, J.I., and Tabata, Y. (2012). Preparation of biodegradable gelatin nanospheres with a narrow size distribution for carrier of cellular internalization of plasmid DNA. J. Biomater. Sci. Polym. Ed. *23*, 991–1004.

44. Miscianinov, V., Martello, A., Rose, L., Parish, E., Cathcart, B., Mitić, T., Gray, G.A., Meloni, M., Al Haj Zen, A., and Caporali, A. (2018). MicroRNA-148b targets the TGF-β pathway to regulate angiogenesis and endothelial-to-mesenchymal transition during skin wound healing. Mol. Ther. *26*, 1996–2007.

45. Umehara, T., Mori, R., Mace, K.A., Murase, T., Abe, Y., Yamamoto, T., and Ikematsu, K. (2019). Identification of specific miRNAs in neutrophils of type 2 diabetic mice: Overexpression of miRNA-129-2-3p accelerates diabetic wound healing. Diabetes *68*, 617–630.

46. Zhang, J., Yang, C., Wang, C., Liu, D., Lao, G., Liang, Y., Sun, K., Luo, H., Tan, Q., Ren, M., and Yan, L. (2016). AGE-induced keratinocyte MMP-9 expression is linked to TET2-mediated CpG demethylation. Wound Repair Regen. *24*, 489–500.

47. Liu, J., Zhang, T., Zhu, J., Ruan, S., Li, R., Guo, B., and Lin, L. (2021). Honokiol attenuates lipotoxicity in hepatocytes via activating SIRT3-AMPK mediated lipophagy. Chin. Med. *16*, 115.

48. Chen, J.L., Feng, Z.L., Zhou, F., Lou, R.H., Peng, C., Ye, Y., and Lin, L.G. (2023). 14-Deoxygarcinol improves insulin sensitivity in high-fat diet-induced obese mice via mitigating NF-κB/Sirtuin 2-NLRP3-mediated adipose tissue remodeling. Acta Pharmacol. Sin. *44*, 434–445.

49. Li, D., Yang, C., Zhu, J.Z., Lopez, E., Zhang, T., Tong, Q., Peng, C., and Lin, L.G. (2022). Berberine remodels adipose tissue to attenuate metabolic disorders by activating sirtuin 3. Acta Pharmacol. Sin. *43*, 1285–1298.

OMTN, Volume *34*

Supplemental information

Exosomal miRNA-155-5p from M1-polarized macrophages suppresses angiogenesis by targeting GDF6 to interrupt diabetic wound healing

Ruohan Lou, Jiali Chen, Fei Zhou, Tian Zhang, Xiuping Chen, Chunming Wang, Bing Guo, and Ligen Lin

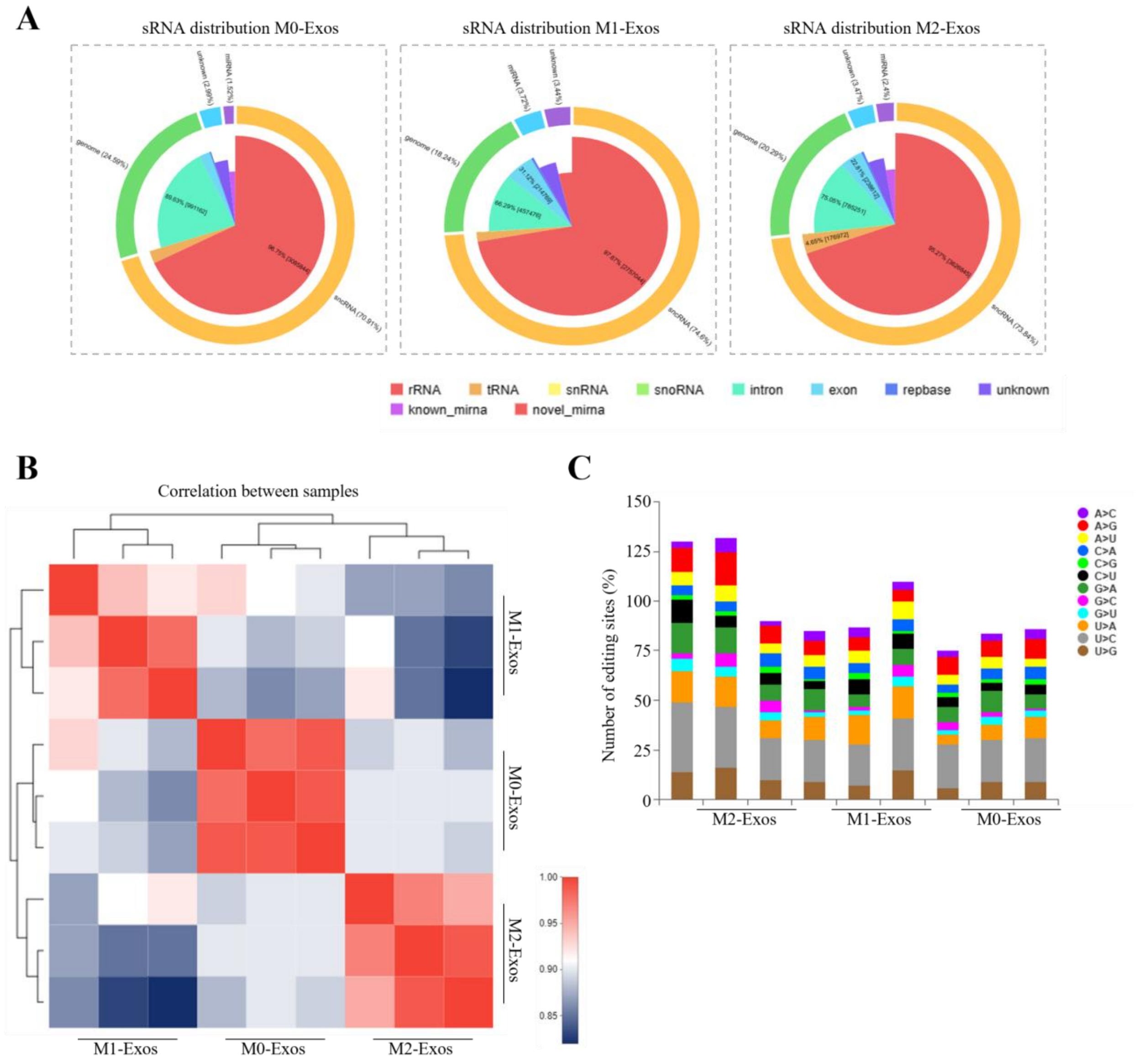

Figure S1. Bioinformatics analysis of exosomal miRNAs. (A) sRNA distribution in exosomes from different groups of mice. (B) Heat map of correlation between samples. (C) Distribution map of miRNA base editing types.

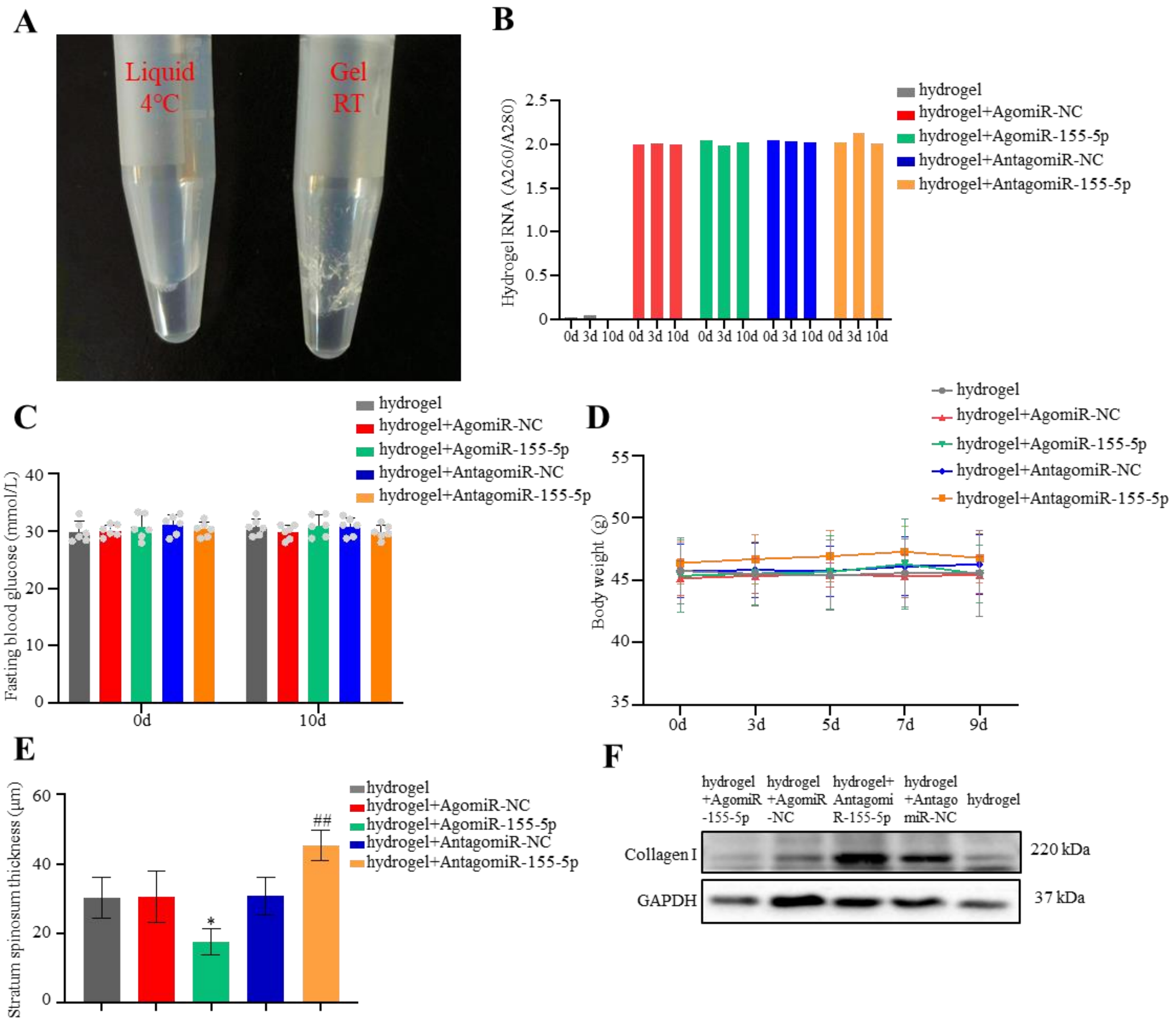

Figure S2. Hydrogel preparation and animal experiments. (A) 25% Pluronic F-127 solution is a liquid at 4 °C and a gel at room temperature. (B) RNA purity in hydrogel. (C) Blood glucose level of *db/db* mice on day 0 and day 10. (D) Body weight of *db/db* mice. (E) Stratum spinosum thickness in wound tissue. (F) The protein expression of collagen I in wound tissue. GAPDH was used as a loading control. $^{*}p < 0.05$, AgomiR-155-5p *vs.* AgomiR-NC; $^{\#\#}p < 0.01$, AntagomiR-155-5p *vs.* AntagomiR-NC.

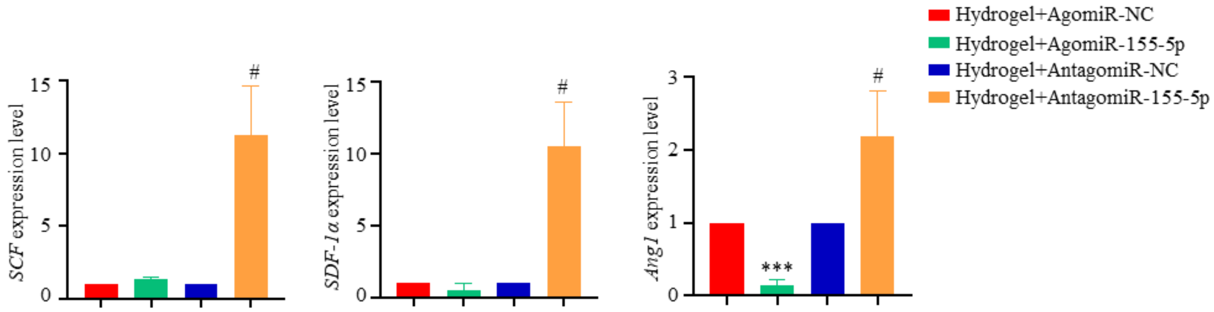

Figure S3. The mRNA expression levels of angiogenic genes in wound tissues were analyzed by q-RT-PCR. ***$p < 0.001$, AgomiR-155-5p *vs.* AgomiR-NC; # $p < 0.05$, AntagomiR-155-5p *vs.* AntagomiR-NC.

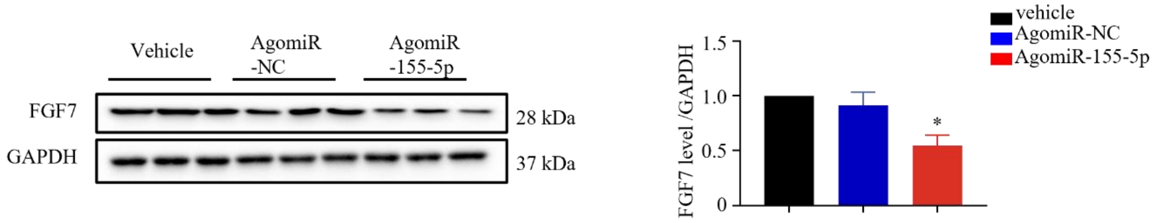

Figure S4. The protein expression of FGF7 in wound tissues. GAPDH was used as a loading control. Data are expressed as means ± SD. $^{*}p < 0.05$, AgomiR-155-5p *vs.* AgomiR-NC.

Table S1

Levels of BMDM-Exos miRNAs and top 10 differentially expressed miRNAs in M1-Exos and M2-Exos.

Table S2

Primers used for RT-PCR.

Names of microRNAs and genes	Primer sequence (5′ to 3′)
has-β-actin-forward	AGAGCTACGAGCTGCCTGAC
has-β-actin-reverse	AGCACTGTGTTGGCGTACAG
has-Bcl-2-forward	GATAACGGAGGCTGGGATGC
has-Bcl-2-reverse	TCACTTGTGGCCCAGATAGG
has-Bax-forward	CCCTTTTGCTTCAGGGTTTC
has-Bax-reverse	GAGACACTCGCTCAGCTTCTTG
hsa-CyclinD1-forward	TTGCCCTCTGTGCCACAGAT
hsa-CyclinD1-reverse	TCAGGTTCAGGCCTTGCACT
hsa-CyclinD3-forward	TACCCGCCATCCATGATCG
hsa-CyclinD3-reverse	AGGCAGTCCACTTCAGTGC
hsa-miRNA-155-5p-RT	CTCAACTGGTGTCGTGGAGTCGGCAATTCAGTTGAGAACCCCTA
has-miRNA-155-5p-forward	GCCGAGTTAATGCTAATCGTG
has-miRNA-155-5p-reverse	CTCAACTGGTGTCGTGGA
